# Virtual Reality–Based Exercise Rehabilitation in Cancer-Related Dysfunctions: Scoping Review

**DOI:** 10.2196/49312

**Published:** 2024-02-26

**Authors:** Zhenzhen Su, Liyan Zhang, Xuemin Lian, Miaomiao Guan

**Affiliations:** 1 School of Nursing Peking University Beijing China; 2 Key Laboratory of Carcinogenesis and Translational Research (Ministry of Education/Beijing) Department of Gastrointestinal Oncology Peking University Cancer Hospital & Institute Beijing China

**Keywords:** virtual reality, cancer, virtual reality–based exercise rehabilitation, cancer-related dysfunction, rehabilitation, scoping review

## Abstract

**Background:**

Virtual reality–based exercise rehabilitation (VRER) is a promising intervention for patients with cancer-related dysfunctions (CRDs). However, studies focusing on VRER for CRDs are lacking, and the results are inconsistent.

**Objective:**

We aimed to review the application of VRER in patients with CRDs.

**Methods:**

This scoping review was conducted following the PRISMA-ScR (Preferred Reporting Items for Systematic Reviews and Meta-Analyses extension for Scoping Reviews) checklist framework. Publications were included from the time of database establishment to October 14, 2023. The databases were PubMed, Embase, Scopus, Cochrane, Web of Science, ProQuest, arXiv, IEEE Xplore, MedRxiv, CNKI, Wanfang Data, VIP, and SinoMed. The population included patients with cancer. A virtual reality (VR) system or device was required to be provided in exercise rehabilitation as an intervention. Eligible studies focused on VRER used for CRDs. Study selection and data extraction were performed by 2 reviewers independently. Extracted data included authors, year, country, study type, groups, sample size, participant age, cancer type, existing or potential CRDs, VR models and devices, intervention programs and durations, effectiveness, compliance, satisfaction, and safety.

**Results:**

We identified 25 articles, and among these, 12 (48%) were randomized clinical trials, 11 (44%) were other experimental studies, and 2 (8%) were observational studies. The total sample size was 1174 (range 6-136). Among the 25 studies, 22 (88%), 2 (8%), and 1 (4%) included nonimmersive VR, immersive VR, and augmented reality, respectively, which are models of VRER. Commercial game programs (17/25, 68%) were the most popular interventions of VRER, and their duration ranged from 3 to 12 weeks. Using these models and devices, VRER was mostly applied in patients with breast cancer (14/25, 56%), leukemia (8/25, 32%), and lung cancer (3/25, 12%). Furthermore, 6 CRDs were intervened by VRER, and among these, postmastectomy syndromes were the most common (10/25, 40%). Overall, 74% (17/23) of studies reported positive results, including significant improvements in limb function, joint range of motion, edema rates, cognition, respiratory disturbance index, apnea, activities of daily living, and quality of life. The compliance rate ranged from 56% to 100%. Overall, 32% (8/25) of studies reported on patient satisfaction, and of these, 88% (7/8) reported satisfaction with VRER. Moreover, 13% (1/8) reported mild sickness as an adverse event.

**Conclusions:**

We found that around half of the studies reported using VRER in patients with breast cancer and postmastectomy dysfunctions through nonimmersive models and commercial game programs having durations of 3-12 weeks. In addition, most studies showed that VRER was effective owing to virtualization and interaction. Therefore, VRER may be an alternate intervention for patients with CRDs. However, as the conclusions were drawn from data with acknowledged inconsistencies and limited satisfaction reports, studies with larger sample sizes and more outcome indictors are required.

## Introduction

### Use of Traditional Exercise Rehabilitation in Patients With Cancer-Related Dysfunctions

Cancer is a leading cause of death, and cancer and its treatments cause varying degrees of cancer-related dysfunctions (CRDs) [1-3]. Studies have shown that the probability of CRDs in pediatric patients is close to 20%, whereas the incidence of CRDs in adult patients exceeds 50% [4]. Common CRDs include chemotherapy-induced peripheral neuropathy (CIPN), sexual dysfunction, cancer-related fatigue (CRF), cancer-related sleep disorder, cancer-related cognitive impairment (CRCI), postoperative syndromes in breast cancer, and cardiopulmonary dysfunction, with incidence rates of up to 96% [5], 95% [6,7], 90% [8], 90% [9], 83% [10], 60% [11], and 28% [12], respectively. These CRDs significantly reduce the quality of life (QoL) of patients and increase the economic burden on the health care system. Exercise rehabilitation, including aerobic, resistance, flexibility, and neuromuscular training, has been widely discussed as a treatment method [13]. It has been shown that exercise rehabilitation can significantly improve CRDs [14-18]. Palm et al [18] demonstrated that exercise rehabilitation, such as physical exercises and pelvic floor muscle exercises, could improve sexual function. Zimmer et al [19] reported the same results, showing a significant alleviation of CIPN after an 8-week exercise rehabilitation program that included endurance, resistance, and balance training [19].

However, traditional exercise rehabilitation has limitations. First, most patients with cancer have few opportunities to participate in exercise rehabilitation. A study on the unmet needs of patients with gynecologic cancers found that only one-third of these patients had access to an exercise rehabilitation program [20]. In addition, 86% of pediatric embryonal brain cancer survivors in Norway had unmet rehabilitation needs [21]. Second, patients with cancer have poor compliance with exercise rehabilitation. Because exercise rehabilitation was performed over a long period, was monotonous, and lacked supervision, up to 50% of patients could not continue their recovery, even after they arrived at the rehabilitation center [22]. Third, exercise rehabilitation programs must be individualized to the characteristics of each patient, which poses a challenge to medical staff [23]. Therefore, research exploring new exercise rehabilitation measures to improve CRDs and the QoL of patients with cancer is urgently needed. In addition, such research will reduce the burden on the medical system. These exercise rehabilitation measures must also ensure patient compliance and satisfaction.

### Use of Virtual Reality–Based Exercise Rehabilitation in CRDs

Virtual reality (VR) is a technology that integrates visual and auditory stimuli through devices such as head-mounted displays, virtual headsets, and virtual glasses. While wearing a VR device, users can interact with the virtual environment through hand controllers and sensors [24]. Four main models of VR systems are commonly used in the medical field: desktop VR, immersive VR, augmented reality (AR), and distributed VR [25]. Desktop VR, also known as nonimmersive VR, allows users to interact with the virtual environment through devices such as a keyboard, mouse, joystick, or touch screen. Immersive VR systems temporarily isolate users from the real world. Users are immersed in virtual environments using interactive devices, such as head-mounted displays, which affect their visual, auditory, and other senses. AR enhances VR by superimposing virtual objects onto the real world to create a more realistic experience. Finally, distributed VR systems connect virtual environments from different locations across the internet, enabling users in multiple locations to participate in the same virtual space and interact effectively. The advancement of technology has enabled the gradual application of VR in patients.

Virtual reality–based exercise rehabilitation (VRER) is a promising intervention that combines VR and exercise rehabilitation. VRER uses VR to create a 3D environment coupled with body tracking to provide safe and realistic scenes [26]. Studies have suggested that VRER effectively improves dysfunctions in patients with Parkinson disease, stroke, and cardiovascular disease. In patients with Parkinson disease, VR technology can provide a virtual scenario with gait and balance rehabilitation, in which patients can engage in multisensory VRER to improve their dysfunctions [27]. In addition, this research found that VRER interventions improved the limb function and walking ability of patients with stroke, through various devices for motor or balance exercises. The improvement of both outcomes exceeded that of traditional exercise rehabilitation [28]. Similarly, patients with cardiovascular disease also benefited from VRER through a series of exergames. The treatment improved participants’ symptoms and cardiorespiratory fitness [29]. Abbas et al [30] proposed that VR could achieve desired rehabilitation outcomes without a therapist, which would significantly reduce rehabilitation costs. Furthermore, VRER is beneficial when traditional rehabilitation services are inadequate or rehabilitation environments are unsafe [30].

### Research Gaps and Aims

As mentioned above, traditional exercise rehabilitation is considered to be boring, lacks accessibility, has low compliance, and lacks individualization. There is an urgent need to improve the rehabilitation effectiveness in patients with CRDs. VRER, as a promising intervention, aimed to overcome the shortage of traditional exercise rehabilitation. Original studies have investigated its application and have reported the models, contents, effectiveness, and other outcomes, but the results of the available research have been inconsistent [24,31]. With the increasing number of VRER interventions, it is important to aggregate the current research via a scoping review, so that researchers can review the applications and limitations of VRER in CRDs.

We searched the Cochrane Database and PubMed for previous systematic reviews and scoping reviews, but no comprehensive reviews were found. Therefore, we conducted a scoping review on the application of VRER in CRDs, with the aim to assess the following: (1) models and contents of VRER in CRDs; (2) types of cancers and CRDs in VRER; (3) effectiveness of VRER; and (4) patient compliance, satisfaction, and safety of VRER in CRDs.

## Methods

### Protocol

A scoping review was conducted to explore the relevant articles on the effectiveness of VRER in CRDs. To conduct and report this scoping review, we followed the PRISMA-ScR (Preferred Reporting Items for Systematic Reviews and Meta-Analyses extension for Scoping Reviews) checklist framework [32,33], but modified some parts to fit our review. The PRISMA-ScR checklist of our review can be found in Multimedia Appendix 1. The protocol details can be found in Multimedia Appendix 2 [4,14-18,20-24,27,29-31]. A scoping review is an ideal method for reporting because it provides comprehensive information about studies of interest [34]. It lists how the study is conducted, defines certain concepts and characterizations, pinpoints key factors or essential issues, analyzes insufficient information, and examines the future direction of a specific field [34]. We chose the scoping review approach as it was suitable for our aims.

### Eligibility Criteria

The PICOS (Population, Intervention, Comparator, Outcome, Study design) framework was used to clarify our search eligibility criteria (Textbox 1). The population involved patients with cancer. The interventions included exercise rehabilitation through VR systems or devices. There were no restrictions on comparators applied. The eligible studies were focused on VRER used for the recovery of CRDs to assess effectiveness or feasibility, and patient satisfaction, compliance, or safety. In addition, the contents of interventions of VRER were required to be displayed. Experimental studies or observational studies with full text in English or Chinese were included, while qualitative research or other such types were excluded because we attempted to explore quantitative outcomes. Publications from the establishment of the database to October 14, 2023, were considered for inclusion.

**PICOS (Population, Intervention, Comparator, Outcome, Study design) framework with the inclusion and exclusion criteria, language, and time limit.**

**Inclusion criteria**

- Population (P): Patients with cancer
- Intervention (I): Exercise rehabilitation through a virtual reality (VR) system or device
- Comparator (C): No comparator was needed. However, when studies were designed with a comparator, such as a control group, it was required to be another intervention or a blank control, and to have baseline data.
- Outcome (O): Feasibility indicators of virtual reality–based exercise rehabilitation (VRER); Effectiveness of VRER; Patient satisfaction, compliance, or safety of VRER
- Study design (S): Experimental design (randomized clinical trial [RCT], quasi-RCT, non-RCT, and before-after study); Observational design
- Language: English or Chinese
- Time limit: From the date of database establishment to October 14, 2023

**Exclusion criteria**

- Population (P): Dysfunction not caused by cancer or its therapy; Not cancer patients
- Intervention (I): No exercise rehabilitation; Exercise rehabilitation without VR; No description of VRER contents
- Comparator (C): No restrictions on the comparators applied
- Outcome (O): Not reported
- Study design (S): Review meeting comment, letter, and editorial protocol only
- Invention: Case report; Guidelines; Qualitative research
- Language: Not in English or Chinese
- Time limit: Time not mentioned in the inclusion criterion

### Information Sources

We searched both English and Chinese databases to identify relevant studies in this review. The databases included PubMed, Embase, Scopus, Cochrane, Web of Science, ProQuest, arXiv, IEEE Xplore, MedRxiv, CNKI, Wanfang Data, VIP, and SinoMed. The databases of arXiv, IEEE Xplore, and MedRxiv were searched because they are professional computer technology databases. Moreover, we searched reference lists to explore further studies of interest.

### Search Strategy

We used a combination of Medical Subject Healings (MeSH) terms and free words to build our strategy in order to achieve perfection and optimization. Two researchers (ZS and XL) independently constructed the search terms based on the PICOS framework and previous research. If their opinions differed, the search terms were decided by another author (LZ). A specific search strategy for PubMed is listed in Textbox 2. The search terms of all databases are presented in Multimedia Appendix 3.

**Specific search strategy for PubMed.**

- #1 “Neoplasms”[MeSH] OR “Cancer”[Title/Abstract] OR “Neoplas*”[Title/Abstract] OR “Carcinoma”[Title/Abstract] OR “Tumo*”[Title/Abstract] OR “Adenocarcinoma”[Title/Abstract] OR “Malignan*”[Title/Abstract]
- #2 “Virtual Reality”[MeSH] OR (“Virtual”[Title/Abstract] AND “Reality”[Title/Abstract])
- #3 “Virtual Reality Exposure Therapy”[MeSH] OR “Exergaming”[MeSH] OR “Exercise Therapy”[MeSH] OR “Exercise”[MeSH] OR “Sports”[MeSH] OR (“Reality Therap*”[Title/Abstract] AND “Virtual”[Title/Abstract]) OR “Active-Video Gaming*”[Title/Abstract] OR “Exergam*”[Title/Abstract] OR “Rehabilitation Exercise*”[Title/Abstract] OR “Remedial Exercise*”[Title/Abstract] OR “Exercise*”[Title/Abstract] OR “Athletic*”[Title/Abstract] OR “Training”[Title/Abstract]
- #4 #1 AND #2 AND #3

### Selection of Sources of Evidence

Two researchers (ZS and MG) independently screened and cross-checked the literature. If their opinions differed, the search terms were decided by another researcher (LZ). All search results were exported to EndNote X9 (Clarivate), and then, we screened and eliminated duplicate articles manually with the software. The study selection process involved 2 steps. First, we eliminated irrelevant studies by reading the titles and abstracts. Second, we read the full text of the remaining studies to identify studies for inclusion (same 2 researchers).

### Data Extraction and Result Synthesis

A data extraction form was developed by ZS and reviewed by LZ. The extracted data were all from full-text studies. The extracted contents included metadata (authors, year, country, study type, groups, sample size, and age), features of VRER application in CRDs (cancer type, existing or potential CRDs, VR models and devices, intervention programs, and duration), effectiveness, satisfaction, compliance, and safety. Two researchers (ZS and XL) independently performed data extraction and cross-checking. If their opinions differed, the search terms were decided by another researcher (LZ).

## Results

### Selection of Evidence

**Figure 1:**
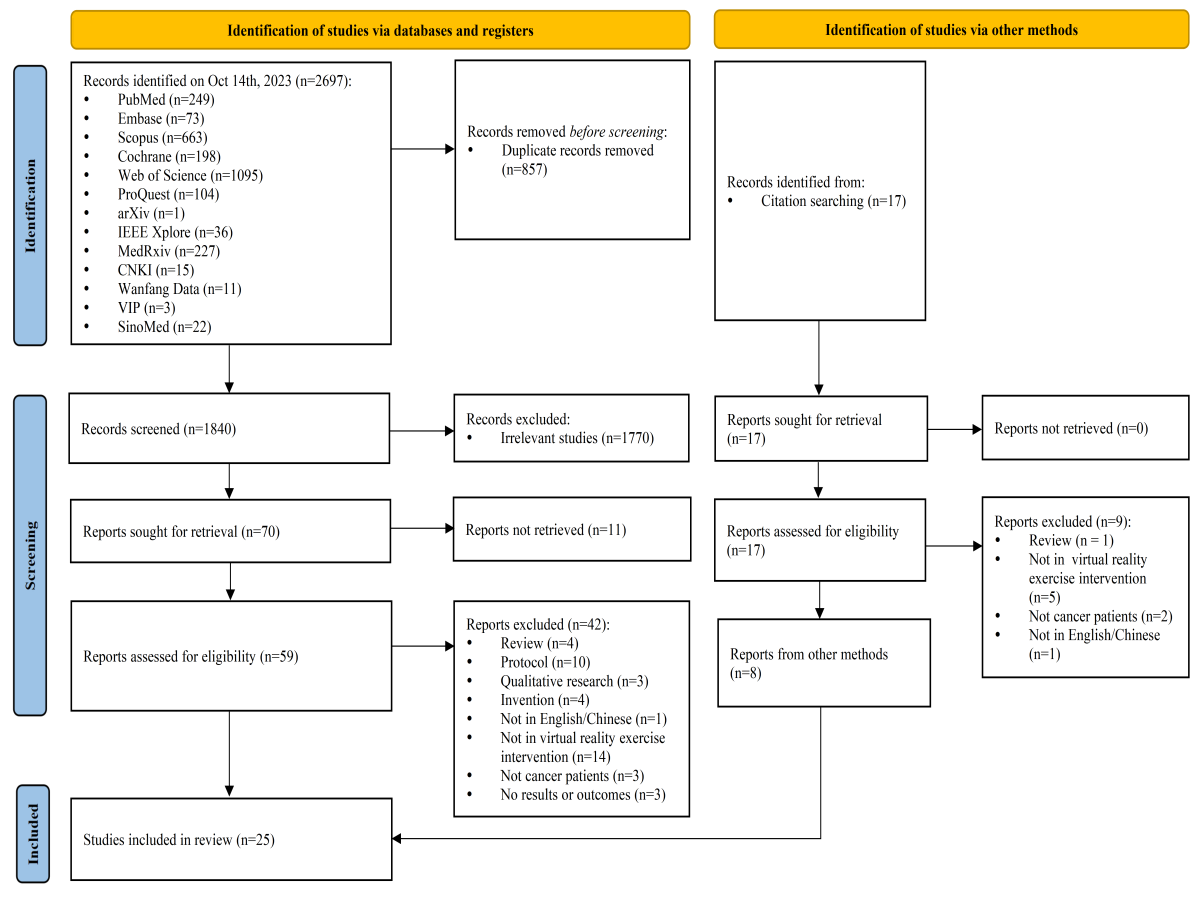
PRISMA-ScR (Preferred Reporting Items for Systematic Reviews and Meta-Analyses extension for Scoping Reviews) flowchart of the study selection process.

The search was conducted from the date of database establishment to October 14, 2023. A total of 2697 English and Chinese records were retrieved from 13 databases, including PubMed, Embase, Scopus, Cochrane, Web of Science, ProQuest, arXiv, IEEE Xplore, MedRxiv, CNKI, Wanfang Data, VIP, and SinoMed. All forms were included in EndNote X9, and 857 duplicate articles were manually removed. The titles and abstracts of 1840 reports were read, and then, 1770 irrelevant reports were excluded. The reasons for exclusion were research at cellular and molecular levels, inconsistent research type and patient type, and VR applications in auxiliary medical methods. After screening the titles and abstracts, 70 reports were included in the full-text screening, and 11 reports were excluded because the full text of the articles was unavailable. Moreover, 42 reports were excluded after reading the full text because the reports were reviews (n=4), protocols (n=10), qualitative studies (n=3), or invention reports (n=4); were not in English or Chinese (n=1); did not address VRER (n=14); did not involve cancer patients (n=3); and did not report results or outcomes (n=3). In addition, we simultaneously conducted a citation search of interest, generating a total of 17 records. Of these, 9 reports were excluded because the reports were reviews (n=1), did not address VRER (n=5), did not involve cancer patients (n=2), and were not in English or Chinese (n=1). Through database and citation searches, 17 and 8 articles were included, respectively. A total of 25 articles ultimately met the predefined inclusion and exclusion criteria and were cited for data extraction [35-59]. Details of the screening process and the included and excluded articles at each stage are shown in the PRISMA-ScR flowchart (Figure 1). Multimedia Appendix 4 shows the data extracted from all 25 studies [35-59], which we will also introduce in the following text.

### Characteristics of the Sources of Evidence

The general characteristics of the included studies are displayed in Table 1. It can be seen that primary published research on VRER dates back as far as 2013. Most studies (12/25, 48%) were randomized clinical trials (RCTs), followed by before-after studies (8/25, 32%), quasi-RCTs (2/25, 8%), observational studies (2/25, 8%), and non-RCTs (1/25, 4%). The total sample size was 1174, ranging from 6 to 136. The results showed that most (10/25, 40%) studies had fewer than 30 participants. The second largest sample size was between 31 and 50 (7/25, 28%). Less than one-third of studies (8/25, 32%) had over 50 participants.

**Table 1:** The time frame, study type, and sample size of the included studies.

Variable		Value (N=25), n (%)	References
**Study year**			
	2013-2018	25 (100)	[35-59]
**Study type**			
	RCT^a^	12 (48)	[36,37,41,42,46,48-51,54,55,59]
	Quasi-RCT	2 (8)	[35,47]
	Non-RCT	1 (4)	[56]
	Before-after study	8 (32)	[38-40,43-45,52,53]
	Observational study	2 (8)	[57,58]
**Sample size**			
	≤30	10 (40)	[35,43-45,50-53,57,58]
	31-50	7 (28)	[39-42,54-56]
	51-80	5 (20)	[36-38,47,59]
	≥81	3 (12)	[46,48,49]

^a^RCT: randomized clinical trial.

### Models and Contents of VRER in CRDs

We explored the models and devices of VR (Table 2). The results showed that nonimmersive VR was the most popular model chosen by researchers (22/25, 88%), while immersive VR and AR were used in 8% (2/25) and 4% (1/25) of included studies, respectively. Regarding VR devices, most studies (16/25, 64%) used commercial devices manufactured by Nintendo, Microsoft, or other technology companies (eg, Nintendo Wii, Xbox Kinect, UINCARE Home+, and IREX system), while 36% (9/25) of studies used self-built VR devices.

**Table 2:** Models and devices of virtual reality systems in the included studies.

Variable		Value (N=25), n (%)	References
**VR^a^ models**			
	Nonimmersive VR	22 (88)	[35-48,50-56,59]
	Immersive VR	2 (8)	[57,58]
	Augmented reality	1 (4)	[49]
**VR device**			
	Nintendo Wii	7 (28)	[35,42-44,50,52,53]
	Xbox Kinect	6 (24)	[36,37,39-41,54]
	Self-built VR	9 (36)	[38,45-48,51,57-59]
	UINCARE Home+	1 (4)	[49]
	IREX system	2 (8)	[55,56]

^a^VR: virtual reality.

Regarding the contents of VRER, we identified commercial game programs and individualized exercise programs. Most (17/25, 68%) of the studies adopted commercial game programs through commercial VR devices like Nintendo Wii and Xbox Kinect, while around one-third (8/25, 32%) of the studies applied individualized exercise programs, with 88% (7/8) using self-built systems (Table 3).

**Table 3:** Programs and durations of virtual reality–based exercise rehabilitation in cancer-related dysfunctions.

Author, year	Contents	
	Programs	Duration
Atef et al [35], 2020	Tennis, triceps extension, and rhythmic boxing	Two sessions per week for 4 weeks
Basha et al [36], 2022	“Macarena” dance and others like darts, bowling, boxing, table tennis, fruit ninja, and beach volleyball	Once per day, 5 times a week for 8 weeks
Benzing et al [37], 2020	Six different “workouts” such as “Waterfall Jump” (jumps onto wood without falling down) and “Derby Skate” (like dance, remember movements, and imitation)	Three times a week for approximately 45 minutes, for 8 weeks
Chen et al [38], 2019	Perform “Tai Chi,” “Cake Cutting,” “Rowing,” “Virtual Shopping,” and other trainings	Once per day, 5 times a week for 8 weeks
da Silva Alves et al [39], 2018	Three games: “Wall Breaker” (hit projected cubes), “Stomp It” (hit lights by moving limbs), and “Run the World” (simulate walking using knee flexion and hip turn movements)	About 45 minutes per session, 3 sessions per week for 8 weeks.
da Silva Alves et al [40], 2017	Three games: “Wall Breaker” (hit projected cubes), “Stomp It” (hit lights by moving limbs), and “Run the World” (simulate walking using knee flexion and hip turn movements)	About 45 minutes per session, 3 sessions per week for 8 weeks
Feyzioĝlu et al [41], 2020	Darts, bowling, and boxing for the first 3 weeks, and beach volleyball, table tennis, and “Fruit Ninja” for the last 3 weeks.	45 minutes per session, 2 sessions a week for 6 weeks
Hamari et al [42], 2019	Various Nintendo Wii games	30 minutes a day, 7 days a week for 8 weeks
Hoffman et al [43], 2013	Walking, balance exercises, including downhill skiing, soccer, golf, and video game activities	30 minutes a day, 5 days a week for 6 weeks
Hoffman et al [44], 2014	Walking, balance exercises, including downhill skiing, soccer, golf, and video game activities	30 minutes a day, 5 days a week for 10 weeks
House et al [45], 2016	Nine custom games: “Breakout 3D” (bounce a virtual ball by paddle avatars), “Card Island,” “Remember that Card” (match card), “Musical Drums” (strike notes using drum stick avatars), “Xylophone” game (repeat notes using mallet avatars), “Pick & Place” (grasp and move balls), “Arm Slalom” (rotate the shoulder to guide a skier avatar through a downhill slalom), “Avalanche” (control a pick axe and a shovel avatar to break and clear ice walls), and “Treasure Hunt” game (control shovel avatars to find buried treasure)	20 to 50 minutes per session, 2 sessions per week for 8 weeks
Jin et al [46], 2018	Breath and raise the leg, ball throw, hair comb, pendulum, shoulder shrug, arm rotation, hand raise, chest expansion, jump, and rollers	30 minutes per session, 1 session per day, 6 days per week for 3 months
Jin et al [47], 2018	Four phases: Phase 1 (1-7 days after surgery): finger strengthening, ball hold and squeeze, knead, and fingertip rub; Phase 2 (8-14 days after surgery): move like a pendulum shrug, wall climb, upper limb movement, and arm rotation; Phase 3 (15 days to 1 month after surgery): flying, rope swing, ball throw, dumbbell exercise, and elastic band; Phase 4 (1-3 months after surgery): hand shake, arm extension, waist turn, and circle exercise	15 to 30 minutes per session, 2 sessions per day for 3 months
Lin et al [48], 2021	Four phases: Phase 1 (1 week after surgery): finger stretch, ball squeeze, ball hold, and fingertip rub; Phase 2 (1-2 weeks after surgery): upper arm movement, body rotation, shoulder lift, shoulder shrug, wall climb, arm swing, and others; Phase 3 (3-4 weeks after surgery): contraction, lateral push and pull, hand swing, chest expansion, lateral lift, circumference, abdominal and back muscle training, lifts, body turns, and others; Phase 4 (5-8 weeks after surgery): head shake, arm extension, waist turn, and circle exercise	For 8 weeks
Park et al [49], 2023	Two parts: Part 1 included passive and active forward flexion, external rotation and abduction, and trunk rotation with both shoulder forward flexion; Part 2 added exercises with dumbbell and pectoralis stretching in addition to part 1.	30 minutes per session, 5 sessions per day, 7 days per week for 8 weeks
Sabel et al [50], 2016	“Sports,” “Sports Resort,” “Fit,” “Fit plus,” “Dance,” and “Michael Jackson Dance”	30 minutes per day, 5 days per week for 10 weeks
Schwenk et al [51], 2016	Three tasks: Balance exercises (forward, backward, sideward, and diagonal leaning tasks); Ankle point-to-point reaching task (forward, backward, sideward, and diagonal leaning and partial weight transfer); Virtual obstacle crossing task (cross obstacles)	45 minutes per session, 2 sessions per week for 4 weeks
Tanriverdi et al [52], 2022	Jogging and hula hoop	45 minutes per session, 2 sessions per day, 2 days per week for 12 weeks
Tsuda et al [53], 2016	Two activities: “Hula Hoop” and “Basic Step” at light to moderate intensity	20 minutes per session, once per day, 5 times a week until hospital discharge
Villumsen et al [54], 2019	Aerobic and strength exercises for 1 hour using “Your Shape Fitness Evolved 2012,” “Sport,” and “Adventure” games	About 1 hour per session, 3 sessions a week for 12 weeks
Yang et al [55], 2014	Five programs: “Conveyor” (move boxes from one side to the other), “Coconut” (catch coconuts in a basket), “Bird and Balls” (catch birds), “Soccer” (stop balls from entering the net), and “Juggler” (hit balls)	30 minutes a day, 3 times a week for 4 weeks
Yoon et al [56], 2015	Six programs: “Birds and Balls,” “Conveyor,” “Drums” (use hands to play a drum along with the music), “Juggler,” “Coconuts,” and “Soccer”	30 minutes per day, 3 days per week for 3 weeks
Zeng et al [57], 2020	Whack-a-mole, golf, fruit cutting, and shooting	NA^a^
Zhou et al [58], 2021	Two models: Model 1 included making fists, rotating the wrists, and bending the elbows; Model 2 included making fists, rotating the wrists, bending the elbows, lifting up, wrapping the shoulders, touching the ears, climbing walls, putting the hands behind the back, holding the head, and abducting	NA
Zhu et al [59], 2019	Four phases: Phase 1 (1 week after surgery): joint functional training like finger stretch, ball squeeze and hold, and fingertip knead; Phase 2 (2 weeks after surgery): shoulder joint abduction, adduction, extension, and forward flexion exercises; Phase 3 (3-4 weeks after surgery): resistance training with dumbbell; Phase 4 (1-3 months after surgery): normal exercise with low intensity	30 minutes per session, 2 sessions per day for 3 months

^a^NA: not available.

The intervention duration of VRER programs was different among studies (Table 3). In the included studies, the programs were conducted for 20 to 60 minutes per session, and 3 to 7 sessions were conducted per week for 3 to 12 weeks. Most were conducted for 8 weeks (9/25, 36%) or 3 months (5/25, 20%). In terms of the time per session, the majority (11/25, 44%) of programs were conducted for 30 minutes per session, and the second most common time was 45 minutes per session.

### Types of Cancers and CRDs Studied in VRER

Table 4 presents the overall general characteristics of the included populations, and types of cancers and CRDs studied in VRER. The population’s age ranged from 3 years to over 70 years. Among all studies, 16% (4/25) reported pediatric cancer patients aged <18 years, who were mainly diagnosed with leukemia, lymphoma, or brain cancer [37,42,50,52]. The remaining 84% (21/25) of studies reported adults with various cancers, including breast cancer, leukemia, lymphoma, brain cancer, nervous system cancer, lung cancer, gastrointestinal tract cancer, abdominal and pelvic cancer, oropharyngeal cancer, multiple myeloma, colorectal cancer, melanoma, bladder cancer, prostate cancer, pancreatic cancer, and ovarian cancer. Among all 25 studies included in the analysis, patients with breast cancer constituted the largest group (14/25, 56%). The second largest cancer type was leukemia (8/25, 32%), and the third largest was lung cancer (3/25, 12%). With these cancers, the most reported CRDs were postmastectomy syndromes, including upper limb dyskinesia and lymphedema (10/25, 40%). The second most reported CRDs were CRCI and CRF (both 5/25, 20%). The third most reported CRDs were extensive physical function damage and neuropathy (3/25, 12%; 1 study about CIPN and 2 studies about cancer-related central neuropathy). Cancer-related sleep disorders (1/25, 4%) were also covered in the included population.

**Table 4:** Population characteristics, types of cancers, and cancer-related dysfunctions studied in virtual reality–based exercise rehabilitation.

Author, year	Age (years), mean (SD) or mean (range)		Cancer type	Types of existing or potential CRDs^a^
	Intervention group	Control group		
Atef et al [35], 2020	54.1 (8.3)	53.1 (7.2)	Breast cancer	Postmastectomy lymphedema
Basha et al [36], 2022	48.8 (7.0)	52.1 (7.5)	Breast cancer	Postmastectomy lymphedema
Benzing et al [37], 2020	11.8 (2.4) and 10.7 (2.5)	11.1 (2.5)	Various types (leukemia, lymphoma, and nervous system cancer)	Central neuropathy
Chen et al [38], 2019	NR^b^	NR	Breast cancer	CRCI^c^
da Silva Alves et al [39], 2018	57.1 (16.7) and 63.3 (7.3)	56.7 (11.9)	Various types (gastrointestinal tract, breast, abdominal, pelvic, and oropharyngeal cancer)	CRF^d^
da Silva Alves et al [40], 2017	57.1 (16.7) and 63.3 (7.3)	56.7 (11.9)	Various types (gastrointestinal tract, breast, abdominal, pelvic, and oropharyngeal cancer)	CRF
Feyzioĝlu et al [41], 2020	50.8 (8.5)	51.0 (7.1)	Breast cancer	Postmastectomy upper limb dyskinesia
Hamari et al [42], 2019	7.8 (range, 3-16)	7.9 (range, 3-15)	Various types (Wilms cancer, acute lymphocytic leukemia, and lymphoma)	Physical function damage and CRF
Hoffman et al [43], 2013	64.6 (range, 53-73)	NR	Lung cancer	CRF
Hoffman et al [44], 2014	64.6 (range, 53-73)	NR	Lung cancer	CRF
House et al [45], 2016	57.8 (20.4)	NR	Breast cancer	CRCI and postmastectomy upper limb dyskinesia
Jin et al [46], 2018	NR	NR	Breast cancer	Postmastectomy upper limb dyskinesia
Jin et al [47], 2018	Range 33-36	Range 35-69	Breast cancer	Postmastectomy upper limb dyskinesia
Lin et al [48], 2021	33.0 (2.8)	33.1 (2.8)	Breast cancer	CRCI
Park et al [49], 2023	42.6 (9.1)	47.3 (8.6)	Breast cancer	Postmastectomy syndrome
Sabel et al [50], 2016	11.9 (3.6)	13.2 (1.9)	Brain cancer	Central neuropathy
Schwenk et al [51], 2016	68.7 (8.7)	71.8 (8.9)	Various types (multiple myeloma, chronic lymphoid leukemia, lung, breast, colorectal, melanoma, bladder, prostate, pancreas, and ovarian cancer)	Chemotherapy-induced peripheral neuropathy
Tanriverdi et al [52], 2022	12.9 (5.8)	NR	Acute lymphoblastic leukemia	Cancer-related sleep disorder
Tsuda et al [53], 2016	66.0 (5.4)	NR	Lymphoma and acute leukemia	Physical function damage
Villumsen et al [54], 2019	67.6 (4.6)	69.8 (4.4)	Prostate cancer	Physical function damage
Yang et al [55], 2014	47.9 (14.5)	52.9 (14.0)	Brain cancer	CRCI
Yoon et al [56], 2015	48.6 (11.3)	50.0 (17.5)	Brain cancer	Postmastectomy upper limb dyskinesia
Zeng et al [57], 2020	NR	NR	NR	CRCI
Zhou et al [58], 2021	54.7 (7.8)	NR	Breast cancer	Postmastectomy upper limb dyskinesia
Zhu et al [59], 2019	58.3 (15.4)	58.6 (15.1)	Breast cancer	Postmastectomy upper limb dyskinesia

^a^CRDs: cancer-related dysfunctions.

^b^NR: not reported.

^c^CRCI: cancer-related cognitive impairment.

^d^CRF: cancer-related fatigue.

### Effectiveness of VRER for CRDs

The effectiveness of VRER for CRDs is shown in Table 5. Two observational studies were excluded in this table because they only observed the current situation without any intervention. The effectiveness included positive (results with significant improvements) and negative results (results with no significant improvements, results without statistical analysis, or inconsistent results). Overall, 74% (17/23) of studies reported positive results. Among the 23 studies, 70% (16/23) reported the results compared with preintervention groups and 65% (15/23) reported the results compared with control groups. Among the studies that reported the results compared with preintervention groups, 75% (12/16) reported positive results after the intervention, including significant improvements in limb function, excessive limb volume, activities of daily living (ADLs), QoL, muscle strength, fear of movement, CRF, balance, body coordination, respiratory disturbance index, apnea, sleep habits, and memory. Conversely, 25% (4/16) of studies reported negative results. Among the studies that reported the results compared with control groups, 60% (9/15) reported positive results after the intervention, including significant improvements in pain intensity, range of motion (ROM) of the joint, fear of movement, edema rate, cognition, ADLs, and QoL.

**Table 5:** Effectiveness of exercise rehabilitation with virtual reality devices (N=23).

Author, year	Results (preintervention or control group vs VRER^a^ group)	
	Compared with preintervention	Compared with control
Atef et al [35], 2020	There were significant improvements in excessive arm volume and affected upper limb dysfunction (9655.27 vs 6854.23 and 55 vs 38, respectively; both *P*<.05). The percentage improvement in excessive arm volume was 26.47% and that of upper limb dysfunction was 33.66%.	No significant differences (*P*>.05).
Basha et al [36], 2022	There were significant improvements in excessive limb volume by 2.63%; disability of the arm, shoulder, and hand by 14.7; ROM^b^ by 25.83°; strength by 1.28 kg; general health by 6.07; mental health by 4.53; vitality by 5.03; physical functioning by 14.7; social functioning by 2.77; and pain by 31.0 (all *P*<.05).	There were significant improvements in pain intensity (53.76 vs 40.33), disability of the arm (27.1 vs 22.9), shoulder ROM (including flexion, abduction, and external rotation, with scores of 126.0 vs 139.0, 102.1 vs 123.3, and 66.1 vs 75.0, respectively), general health (54.9 vs 58.4), and vitality (53.5 vs 55.7) (all *P*<.05).
Benzing et al [37], 2020	NR^c^	No significant differences (*P*>.05).
Chen et al [38], 2019	There were significant improvements in cognitive function (26.22 vs 17.41) and daily life ability (16.05 vs 20.79) (all *P*<.05).	NA^d^
da Silva Alves et al [39], 2018	There were significant improvements in the general functional assessment (88.13 vs 95.37), functional outcomes index (76.00 vs 92.68), and fatigue assessment (121.20 vs 138.99) (all *P*<.05).	NA
da Silva Alves et al [40], 2017	There were significant improvements in maximal voluntary isometric contraction of the right dorsiflexor muscles (6.62 vs 10.9), right plantar flexion (15.01 vs 22.59), and left medial gastrocnemius (158.61 vs 185.23) (all *P*<.05).	NA
Feyzioĝlu et al [41], 2020	There were significant improvements in pain (6.53 vs 1.53), ROM (by 55.39°), muscle strength (5.97 vs 6.25), grip strength (21.47 vs 23.71), functionality (44.67 vs 16.49), and fear scores (42.37 vs 29.47) (all *P*<.05).	Fear of movement was significantly improved by VRER, while the traditional exercise rehabilitation displayed more improvement in functionality (5.94 vs 12.9 and −36.54 vs −28.18, respectively) (both *P*<.05).
Hamari et al [42], 2019	No significant differences (*P*>.05).	No significant differences (*P*>.05).
Hoffman et al [43], 2013	There were improvements in CRF^e^ severity (4.8 vs 2.5), perceived self-efficacy for fatigue self-management (7 vs 8.8), walking and balance (47.4% vs 93.3% and 72.8% vs 83.7%, respectively), and functional performance (steps taken per day, 4650 vs 6393). No statistical analysis.	NA
Hoffman et al [44], 2014	There were improvements in CRF severity (4.8 vs 1.3), perceived self-efficacy for fatigue self-management (7 vs 9), walking and balance (47.4% vs 99.4% and 72.8% vs 88.9%, respectively), and functional performance (steps taken per day, 4650 vs 7683). No statistical analysis.	NA
House et al [45], 2016	There were significant improvements in ROM by 8°, strength by 8.2 N, daily life ability by 13.8, depression by −5.7, and visuospatial memory by 8 (all *P*<.05).	NA
Jin et al [46], 2018	NR	There was a significant improvement in QoL^f^ (75.1 vs 85.4; *P*<.05).
Jin et al [47], 2018	NR	There were significant improvements in shoulder ROM (57.8° vs 81.6°), compliance (84% vs 100%), and edema rate of the affected limbs (42% vs 11%) (all *P*<.05).
Lin et al [48], 2021	NR	There were significant improvements in postoperative complication rates (67% vs 11%), compliance (71% vs 95%), shoulder ROM (57.9° vs 81.6°), daily life ability (20.84 vs 16.07), QoL (15.39 vs 18.69), and cognition (24.60 vs 29.46) (all *P*<.05).
Park et al [49], 2023	There were significant improvements in ROM (109.56 vs 169.12), limb dysfunction (22.05 vs 17.07), and QoL (0.69 vs 0.85) (all *P*<.05).	No significant differences (*P*>.05).
Sabel et al [50], 2016	The body coordination score improved by 15% (*P*<.05).	No significant differences (*P*>.05).
Schwenk et al [51], 2016	NR	There were significant improvements in the medial-lateral center of mass sway (21.2% vs 55.5%), hip sway (42.3% vs 67.5%), and ankle sway (21.4% vs 68.2%) (all *P*<.05).
Tanriverdi et al [52], 2022	There were significant improvements in the Respiratory Disturbance Index (3.10 vs 1.97), number of apnea episodes (15.55 vs 10.14), and sleep habits (57.45 vs 48.27) (all *P*<.05).	NA
Tsuda et al [53], 2016	No significant differences (*P*>.05).	NA
Villumsen et al [54], 2019	NR	There was a significant improvement in the 6-min walking test by 4.2% (*P*<.05).
Yang et al [55], 2014	There were significant improvements in the results of the continuous performance test (0.65 vs 0.55), digit span test (3.9 vs 5.1), visual span test (3.5 vs 4.6), learning test-recognition (30.5 vs 40.7), Trail Making Test-type A (137.9 vs 70.2), Korean version of the Modified Barthel Index (43.4 vs 73.7), and Korean version of the Mini-Mental Status Examination (19.8 vs 25.0) (all *P*<.05).	There were significant improvements in the results of the continuous performance test (−0.2 vs 0.0), backward digit span test (1.4 vs 0.3), backward visual span test (1.4 vs 0.7), and Trail Making Test-type A (−67.7 vs −24.5) (all *P*<.05).
Yoon et al [56], 2015	There were significant improvements in manual dexterity (30.5 vs 38.0), manual function test results (70.3 vs 82.8), Fugl-Meyer scale scores of upper extremity motor ability (52.0 vs 58.0), and activity of life (52.5 vs 70.5) (all *P*<.05).	There were significant improvements in manual dexterity (8.0 vs 11.0), manual function test results (shoulder/elbow/forearm section, 5.0 vs 7.0), and Fugl-Meyer scale scores (shoulder/elbow/forearm section, 2.0 vs 3.5) (all *P*<.05).
Zhu et al [59], 2019	NR	There were significant improvements in compliance (72.5 vs 90), shoulder ROM (57.84° vs 81.65°), and postoperative complication rates (12.5% vs 32.5%) (all *P*<.05).

^a^VRER: virtual reality–based exercise rehabilitation.

^b^ROM: range of motion.

^c^NR: not reported.

^d^NA: not available.

^e^CRF: cancer-related fatigue.

^f^QoL: quality of life.

### Compliance, Satisfaction, and Safety of VRER

Patient-reported compliance, satisfaction, and safety of VRER are presented in Table 6. Some studies did not report some indicators. Compliance and satisfaction rates or scores were either explicitly stated by the authors or calculated from flow charts. If the satisfaction rate was above 85% or the score was over 3.4/4, 4.3/5, or 5.1/6, we defined the patient-reported result as satisfactory. If the satisfaction rate was lower than 60% or the score was less than 2.4/4, 3.0/5, or 3.6/6, we defined the patient-reported result as unsatisfactory.

**Table 6:** Patient compliance, satisfaction, and safety of exercise rehabilitation with virtual reality devices.

Variable		Value (N=25), n (%)
**Compliance (%)**		22 (88)
	100	6 (27)
	85-99	8 (36)
	70-84	6 (27)
	60-69	1 (5)
	<60	1 (5)
**Satisfaction**		8 (32)
	Satisfactory	7 (88)
	Neutral	1 (13)
	Unsatisfactory	0 (0)
**Safety (adverse events)**		8 (32)
	None	7 (88)
	Sickness	1 (13)

As shown in Table 6, 88% (22/25) of studies reported compliance rates, ranging from 56% to 100%. Among them, the majority (13/22, 55%) reported compliance rates between 85% and 99%. Unfortunately, 5% (1/22) reported a compliance rate lower than 60%. With regard to satisfaction, 32% (8/25) of studies provided results. Among them, 88% (7/8) reported satisfaction with VRER and 13% (1/8) reported a neutral result. None of the studies reported unsatisfactory results. With regard to adverse events, 8 studies provided results. Among them, 88% (7/8) reported no adverse events and 12% (1/8) reported mild sickness.

## Discussion

### Principal Findings

VRER is a promising intervention for rehabilitation in patients with CRDs. However, studies focusing on this intervention are lacking and have inconsistent results. In this scoping review, we aimed to explore the applications of VRER used for CRDs, in order to provide a comprehensive summary of studies and to provide a reference for clinical practice.

Of 2714 studies, 25 records were identified that fit our eligibility criteria through screening and analysis. Most of the studies were RCTs (12/25, 48%) and before-after studies (8/25, 32%). The total sample size was 1174, ranging from 6 to 136. According to our objectives, there were 3 categories of results, and each of them provided classification of studies from a different aspect.

First, there were several models and contents of VRER. The models of VR included nonimmersive VR, immersive VR, and AR. Nonimmersive VR was the most (22/25, 88%) used branch through both commercial (16/25, 74%) and self-built devices (9/25, 36%). Within these 3 models, researchers preferred choosing commercial games (17/25, 68%). The other 32% (8/25) of studies applied individualized exercise programs, which were mainly conducted by self-built systems (7/8, 88%). The duration of VRER ranged from 3 to 12 weeks.

Second, there were various types of cancers and CRDs intervened by different VRER approaches. In our review, the most common cancers were breast cancer (14/25, 56%), leukemia (8/25, 32%), and lung cancer (3/25, 12%). Postmastectomy syndromes, including upper limb dyskinesia and lymphedema, were the main CRDs (10/25, 40%).

Third, the effectiveness, compliance, satisfaction, and safety of VRER were inconsistent in the publications. Positive results included significant improvements in limb function, joint ROM, edema rates, cognition, respiratory disturbance index, apnea, ADLs, and QoL. The compliance rate ranged from 56% to 100%. None of the studies reported unsatisfactory results, while 1 study reported mild sickness.

**Figure 2:**
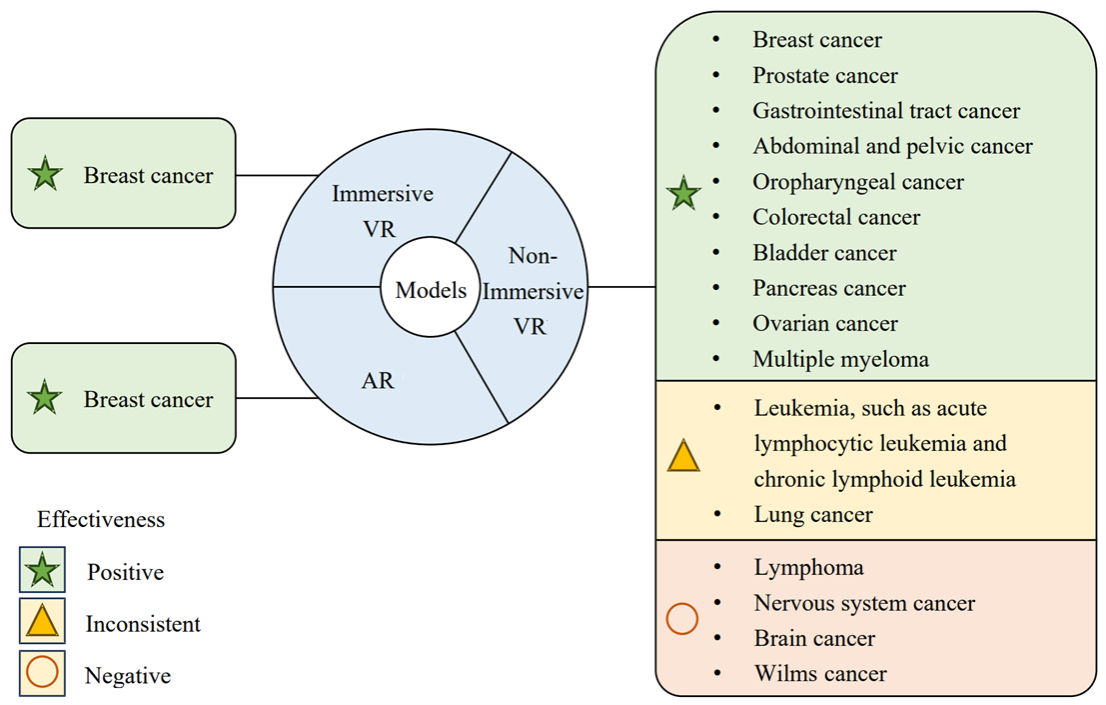
Distribution and efficacy of virtual reality–based exercise rehabilitation applications across different types of cancers. Inconsistent indicates that some studies showed positive results while others showed negative results for the same cancer. AR: augmented reality; VR: virtual reality.

Figure 2 provides an illustration of the distribution and efficacy of VRER applications across different types of cancers.

### Comparison With Prior Work

#### Models and Contents of VRER

In our review, 88% (22/25) of studies applied nonimmersive VR systems for rehabilitation, while the remaining 12% (3/25) applied immersive VR and AR. Among them, the contents were mainly commercial game programs, which were reported in 68% (17/25) of studies. Commercial devices like Nintendo Wii and Xbox Kinect were mostly (13/25, 52%) used. These findings are consistent with the results of other systematic reviews [60]. The reasons for the dominance of nonimmersive VR and commercial game programs include low cost, easy use, and high accessibility [61]. In addition, commercial devices have mature game systems that involve various fun rehabilitation approaches [62].

We also discovered different views from other studies. In our review, nonimmersive VR more frequently led to less adverse events than immersive VR, and thus, people preferred nonimmersive VR [58]. This finding is not consistent with the results in the studies by Tuena et al [63] and Buche et al [64], who found that immersive VR appeared to be associated with less motion sickness. The difference in the findings may be related to the intervention duration. In our review, studies reported that the duration ranged from 3 to 12 weeks, whereas over half of the studies in the review by Tuena et al [63] conducted 1 session of less than 45 minutes. Studies with a larger duration and with controls can be implemented in the future with nonimmersive and immersive VR to obtain definite conclusions.

We noted that over one-third (9/25, 36%) of studies developed self-built VRER systems. Most of them considered the differences in the disease durations and exercise preferences of patients. These studies aimed to improve the outcomes through individual design. Unfortunately, the effectiveness of these systems was compared with that of traditional exercise rehabilitation but not with that of commercial VRER. Further research is needed in this field.

#### Types of Cancers and CRDs in Studies on VRER

We found that around half of the studies on VRER involved patients with breast cancer and postmastectomy dysfunction. This finding is similar to the results in other studies [60,65]. The reasons may be that breast cancer is the most common cancer worldwide and postoperative dysfunction continues from treatment to the survival stage. Thus, there is a need for symptom management, and this area is worthy of attention [66].

Moreover, we identified VRER for several other types of cancers and CRDs. Some studies (13/25, 52%) reported the use of VRER in patients with leukemia, lymphoma, brain cancer, nervous system cancer, lung cancer, gastrointestinal cancer, abdominal and pelvic cancer, oropharyngeal cancer, multiple myeloma, colorectal cancer, melanoma, bladder cancer, prostate cancer, pancreas cancer, and ovarian cancer. Furthermore, research (15/25, 60%) on CRDs, including CRCI, CRF, extensive physical dysfunction, CIPN, cancer-related central neuropathy, and sleep disorders, reported that intervention by VRER was effective. Considering the diversity of cancers and CRDs, we adopted a broader scope to explore the application of VRER in CRDs. However, the number of publications was limited. Future studies should consider a greater number of cancers and CRDs.

#### Effectiveness of VRER

Another significant finding of our study was that VRER can effectively improve CRDs in patients with cancer. Buche et al [64] reviewed VRER application in CRDs and reported a similar result.

**Figure 3:**
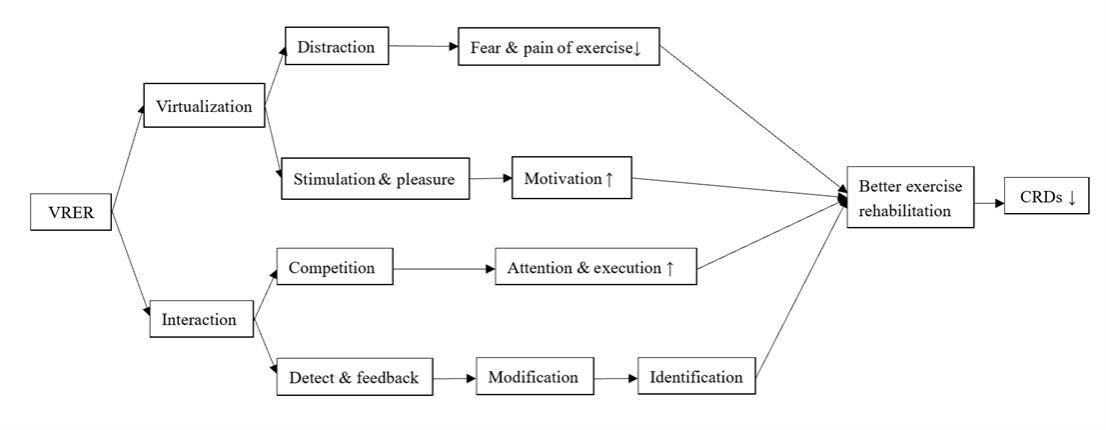
The potential mechanisms of virtual reality–based exercise rehabilitation (VRER) in cancer-related dysfunctions (CRDs).

In addition to postmastectomy syndromes, other types of CRDs could also be intervened effectively by VRER. Studies showed that VRER can improve limb function, joint ROM, edema rates, cognition, respiratory disturbance index, apnea, ADLs, and QoL in cases of CRDs like CRCI, CIPN, and cancer-related sleep disorder [38-40,43-45,48,50-52,54,55]. The potential mechanism was speculated to be closely related to the virtualization and interaction of VR. On one hand, the virtual environment created by VRER could alleviate the fear of movement and the pain of exercise among patients, enabling them to execute exercise rehabilitation better through distraction [36,67]. Moreover, the virtual environment provided stimuli and pleasure, encouraging participants to be more active [68-70]. On the other hand, VR can directly affect exercise rehabilitation through interaction. The interaction associated with VR generates visual stimuli through which patients can identify differences between their movements and the correct ones [71]. Thus, patients’ objective indicators and stimulus feedback allow VR games to be continuously adjusted to achieve superior results [41]. Therefore, it can be more personalized and accurate [38]. In addition, interaction encourages competition and repetition, improving participants’ focus and executive function [72,73]. The potential mechanisms of VRER in CRDs are depicted in Figure 3.

Undeniably, there is no certainty that VRER is better than traditional exercise rehabilitation. Many (10/25, 40%) studies had a small sample size or lacked a randomized and controlled design, which made the results of the studies somewhat different, and the studies lacked sufficient convincing power. We look forward to adding large-sample RCTs in the future.

#### Compliance, Satisfaction, and Safety of VRER

In addition to clinical effectiveness, we found that it is highly feasible for medical staff and patients to use VRER for CRDs. The median compliance rate was 91% (IQ1-IQ3=82%-99%) in the 25 studies, which is much higher than the rate of 71% or less for traditional exercise rehabilitation [74]. Furthermore, high satisfaction and mild VRER adverse events indicated that VRER was widespread and readily accepted. These results were similar to the findings of a systematic review of VR for symptom management in cancer patients, which reported high retention rates in most VR interventions [75]. However, only 8 studies (8/25, 32%) reported on satisfaction, with 88% (7/8) reporting satisfactory results on VRER (satisfaction rate of over 85% or scores of 3.4/4, 4.3/5, or 5.1/6). When we explored the safety of VRER devices, we found that only 1 study (1/8, 13%) reported mild sickness. Although adverse events have been reported less frequently and the reported adverse event was mildly symptomatic, such events may have some impact on patients. As Zhou et al [58] reported, cybersickness is unavoidable in the use of VR owing to the desynchronization of visual stimuli with neural signals. Therefore, when VRER is used, we suggest to introduce it slowly and carefully according to the feedback received from patients. In addition, when reporting outcomes, future studies on VRER for CRDs need to include compliance, satisfaction, and occurrence of adverse events as indicators to better understand the feasibility and safety of VRER.

### Limitations and Future Implications

Our study has some limitations. First, we found that the sample sizes of the studies varied widely, ranging from 6 to 136, which may have introduced variability in our findings. Additionally, more than one-third (10/25, 40%) of the studies had a sample size of less than 30, making it difficult to obtain convincing results. Second, not all studies reported all the indicators we required, which may have reduced the accuracy of our findings, and the validity of the results should be interpreted with caution. In view of the variety of CRDs, VRER application is still relatively limited. Third, there was a trend of using self-built VR systems, but comparisons with other VR systems were lacking. For these reasons, it is difficult to determine the effectiveness of VRER for CRDs, and its adoption should be treated with caution.

Based on these limitations, we have made 3 suggestions for future research. When assessing VRER for CRDs, researchers should (1) increase the sample size to enable more people to use VRER in order to obtain valuable and credible outcomes; (2) include more types of CRDs and more comprehensive indicators like compliance, satisfaction, and safety; and (3) develop more self-built VRER systems with individual content and perform comparisons with traditional VRER systems.

### Conclusion

To the best of our knowledge, this is the first scoping review to provide the most comprehensive data for VRER in patients with CRDs. We summarized the types, models, contents, effectiveness, compliance, satisfaction, and safety of VRER for CRDs, as well as mapped the potential mechanism. Our study found that VRER was mainly used in patients with breast cancer and postmastectomy dysfunctions through nonimmersive models and commercial game programs. Moreover, we found that VRER is an effective intervention accompanied with high compliance and satisfaction for CRDs. However, our findings regarding the effectiveness of VRER are drawn from data with acknowledged inconsistencies and limited satisfaction reports. Thus, it is critical to consider these conclusions with caution. In addition, the sample size, types of CRDs, reported indicators, and VR systems were limited. Nevertheless, we believe that this review can help clinical practices to better understand the applications of VRER for CRDs and to better determine whether to use this approach. We believe that VRER has further unexploited potential in rehabilitation and health care for CRDs, but additional research is needed to solidify these findings. For VRER to be properly accepted in the real word, studies involving larger sample sizes, more CRDs with individual content, and more outcome indictors are required.

## Appendix

Multimedia Appendix 1 PRISMA-ScR (Preferred Reporting Items for Systematic Reviews and Meta-Analyses extension for Scoping Reviews) checklist.

Multimedia Appendix 2 Protocol.

Multimedia Appendix 3 Search terms used to identify studies.

Multimedia Appendix 4 Data extracted from all 25 studies.

## Abbreviations

ADLs: activities of daily living
AR: augmented reality
CIPN: chemotherapy-induced peripheral neuropathy
CRCI: cancer-related cognitive impairment
CRD: cancer-related dysfunction
CRF: cancer-related fatigue
PICOS: Population, Intervention, Comparator, Outcome, Study design
PRISMA-ScR: Preferred Reporting Items for Systematic Reviews and Meta-Analyses extension for Scoping Reviews
QoL: quality of life
RCT: randomized clinical trial
ROM: range of motion
VR: virtual reality
VRER: virtual reality–based exercise rehabilitation

## Data Availability

The data sets generated during this study are available from the corresponding author on reasonable request.
