# Supplementary material for: Virtual Reality–Based Exercise Rehabilitation in Cancer-Related Dysfunctions: Scoping Review
Source: J Med Internet Res. 2024 Feb 26;26:e49312. doi: 10.2196/49312 (PMC10928524; doi:10.2196/49312)
Supplement: Multimedia Appendix 2 [file jmir_v26i1e49312_app2.pdf]

Virtual Reality-Based Exercise Rehabilitation in Cancer-Related Dysfunctions: A Scoping Review Protocol

Abbreviations

CRDs: cancer-related dysfunctions

VRER: virtual reality-based exercise rehabilitation

VR: Virtual reality

CRCI: cancer-related cognitive impairment

CRF: cancer-related fatigue

CIPN: chemotherapy-induced peripheral neuropathy

CRSD: cancer-related sleep disorder

AR: augmented reality

MeSH: Medical Subject Headings

PICOS: Population, Intervention, Comparator, Outcomes, Study design

PRISMA-ScR: Reporting Items for Systematic Reviews and Meta-Analyses extension for Scoping Reviews

M±SD: Mean ± standard deviation

Introduction

VR is a technology that integrates visual and auditory stimuli through devices such as head-mounted displays, virtual headsets, and virtual glasses. While wearing a VR device, users can interact with the virtual environment through hand controllers and sensors [24]. With the increasingly availability and advanced technology, there is expanded usage of in health care in recent years and continues to be investigated.

Cancer-related dysfunctions (CRDs), caused by cancer and related treatments, showed high rates in pediatric (nearly 20%) and adult patients (exceeds 50%) [4].These CRDs significantly reduce the quality of life of patients and increase the economic burden on the healthcare system. Exercise rehabilitation, including aerobic, resistance, flexibility, and neuromuscular training, has been shown effective in improving CRDs [14-18]. However, traditional exercise rehabilitation has limitations with few opportunities, low adherence, and non-individualized [20-23].

VR is a broad concept with many different types of interventions can be developed. Based on this reason, virtual reality-based exercise rehabilitation (VRER) was reported. VRER is a promising intervention that combines virtual reality (VR) and exercise rehabilitation. Studies have suggested that VRER effectively improves dysfunctions in patients with Parkinson's disease, stroke, and cardiovascular disease [27,29,30]. Furthermore, VRER is beneficial when traditional rehabilitation services are inadequate or rehabilitation environments are unsafe [30].

Unfortunately, little research has been carried out on the application of VRER in patients with cancer, and the results of the available research have been inconsistent [24,31]. Therefore, it is essential to explore the possibilities and feasibility in cancer patients.

Objective

We conducted a scoping review to review the application of VRER in CRDs, including 4 aspects:

- The types of cancers and CRDs were studied in VRER;
- Models and contents of VRER in CRDs;
- The effective of VRER;
- The safety, patients` compliance and satisfaction of VRER in CRDs.

We are also going to analyze the mechanism of action through which VRER caused these improvements to provide a reference for clinical oncology practices.

Methods

Eligibility criteria

PICOS (Population, Intervention, Comparator, Outcome, Study design) framework were used to clarify our search eligibility criteria, and lists as follows.

Population (P):

- Inclusion criteria: Cancer patients
- Exclusion criteria: The dysfunction not caused by cancer or it`s therapy. Not cancer patients

Intervention (I)

- Inclusion criteria: Exercise rehabilitation through VR system or device
- Exclusion criteria: Not exercise rehabilitation. Exercise rehabilitation without VR. Not description of VRER contents

Comparator (C)

- No restrictions applied.

Outcome (O)

- Inclusion criteria: The feasibility or safety indicates of VR. Exercise rehabilitation or clinical outcomes
- Exclusion criteria: Not reported

Study design (S)

- Inclusion criteria: Experimental design (quasi-RCTs, RCTs and before-after study in the same patient, after test only without control study). Observational design: feasibility study
- Exclusion criteria: Review, Meeting, Comment, letter and editorial, Protocol only, Invention, Case report, Guidelines, Qualitative research

Language

- Inclusion criteria: English/Chinese
- Exclusion criteria: Not in English/Chinese

Time limit

- Inclusion criteria: From the date of database establishment to Oct 14th, 2023
- Exclusion criteria: Time out of the inclusion criterion

Research sources and strategy

We searched both English and Chinese database to catch needed studies included in this review.

- English database (n=9)

PubMed, Embase, Scopus, Cochrane, Web of Science, ProQuest, arXiv, IEEE Xplore, MedRxiv

- Chinese database (n=4)
  CNKI, Wanfang Data, VIP and SinoMed

Reference lists were also select to explore the further studies of interest.

Taking the form of a combination of Medical Subject Healings (MeSH) terms and free words to build our strategy, so as to make strategy perfect and optimized. Two researchers (Su and Guan) independently constructed the search terms based on the PICOS framework and previous research. If the opinions differed, the search terms were decided by another professor.

The search terms used of all database for the are presented in Table 1 (English database) and Table 2 (Chinese database).

Table 1: Search Terms used of English database

Database	Search Terms	Hits
PubMed	#1 "Neoplasms"[MeSH] OR "Cancer"[Title/Abstract] OR "Neoplas*"[Title/Abstract] OR "Carcinoma"[Title/Abstract] OR "Tumo*"[Title/Abstract] OR "Adenocarcinoma"[Title/Abstract] OR "Malignan*"[Title/Abstract]<br>#2 "Virtual Reality"[MeSH] OR ("Virtual"[Title/Abstract] AND "Reality"[Title/Abstract])<br>#3 "Virtual Reality Exposure Therapy"[MeSH] OR "Exergaming"[MeSH] OR "Exercise Therapy"[MeSH] OR "Exercise"[MeSH] OR "Sports"[MeSH] OR ("Reality Therap*"[Title/Abstract] AND "Virtual"[Title/Abstract]) OR "Active-Video Gaming*"[Title/Abstract] OR "Exergam*"[Title/Abstract] OR "Rehabilitation Exercise*"[Title/Abstract] OR "Remedial	249

	Exercise*"[Title/Abstract] OR "Exercise*"[Title/Abstract] OR "Athletic*"[Title/Abstract] OR "Training"[Title/Abstract]<br>#4 #1 AND #2 AND #3	
Embase	#1 ‘malignant neoplasms’/exp OR ‘cancer’:ti,ab,kw OR ‘neoplas*’:ti,ab,kw OR ‘carcinoma’:ti,ab,kw OR ‘tumo*’:ti,ab,kw OR ‘adenocarcinoma’:ti,ab,kw OR ‘malignan*’:ti,ab,kw<br>#2 ‘virtual reality’/exp<br>#3 ‘exergaming’/exp OR ‘athletic rehabilitation’/exp OR ‘exercise’/exp OR ‘training’/exp OR ‘kinesiotherapy’/exp OR ‘active video gaming’:ti,ab,kw OR ‘exer-gaming’:ti,ab,kw OR ‘virtual reality-based exercise’:ti,ab,kw OR ‘sport rehabilitation’:ti,ab,kw OR ‘corrective exercise’:ti,ab,kw OR ‘exercise therapy’:ti,ab,kw OR ‘exercise movement techniques’:ti,ab,kw OR ‘exercise treatment’:ti,ab,kw OR ‘kinesiotherapeutic’:ti,ab,kw OR ‘kinesitherapeutic’:ti,ab,kw OR ‘therapeutic exercise’:ti,ab,kw OR ‘exertion’:ti,ab,kw OR ‘detraining’:ti,ab,kw<br>#4 #1 AND #2 AND #3	73
Scopus	#1 TITLE-ABS-KEY("Cancer" OR "Neoplas*" OR "Carcinoma" OR "Tumo*" OR "Adenocarcinoma" OR "Malignan*")<br>#2 TITLE-ABS-KEY("Virtual Reality" OR ("Virtual" AND "Reality"))<br>#3 TITLE-ABS-KEY("Virtual Reality Exposure Therapy" OR ("Reality Therap*" AND "Virtual") OR "Exergaming" OR "Exercise Therapy" OR "Exercise" OR "Sports" OR "Rehabilitation Exercise*" OR "Remedial Exercise*" OR "Exercise*" OR "Athletic*" OR "Training" OR "Active-Video Gaming*" OR "Exergam*")	663

	#4 #1 AND #2 AND #3	
Cochrane	#1 (Neoplasms)/exp OR (Cancer):ti,ab,kw OR (Neoplas*):ti,ab,kw OR (Carcinoma):ti,ab,kw OR (Tumo*):ti,ab,kw OR (Adenocarcinoma):ti,ab,kw OR (Malignan*):ti,ab,kw<br>#2 (Virtual Reality)/exp OR ((Virtual):ti,ab,kw AND (Reality):ti,ab,kw)<br>#3 (Virtual Reality Exposure Therapy)/exp OR (Exergaming)/exp OR (Exercise Therapy)/exp OR (Exercise)/exp OR (Sports)/exp OR ((Reality Therap*):ti,ab,kw AND (Virtual):ti,ab,kw) OR (Active-Video Gaming*):ti,ab,kw OR (Exergam*):ti,ab,kw OR (Rehabilitation Exercise*):ti,ab,kw OR (Remedial Exercise*):ti,ab,kw OR (Exercise*):ti,ab,kw OR (Athletic*):ti,ab,kw OR (Training):ti,ab,kw<br>#4 #1 AND #2 AND #3	198
Web of Science	#1 TS=(Cancer OR Neoplas* OR Carcinoma OR Tumo* OR Adenocarcinoma OR Malignan*)<br>#2 TS=(Virtual Reality OR (Virtual AND Reality))<br>#3 TS=(Virtual Reality Exposure Therapy OR (Reality Therap* AND Virtual) OR Exergaming OR Exercise Therapy OR Exercise OR Sports OR Rehabilitation Exercise* OR Remedial Exercise* OR Exercise* OR Athletic* OR Training OR Active-Video Gaming* OR Exergam*)<br>#4 #1 AND #2 AND #3	1095
ProQuest	SU(malignant neoplasms) OR TI,AB(cancer OR neoplas* OR carcinoma OR tumo* OR adenocarcinoma OR malignan*)<br>#2 SU(virtual reality)<br>#3 SU(exergaming OR athletic rehabilitation OR exercise OR training OR kinesiotherapy) OR TI,AB(active video gaming OR	104

	exer-gaming OR virtual reality-based exercise OR sport rehabilitation OR corrective exercise OR exercise therapy OR exercise movement techniques OR exercise treatment OR kinesiotherapeutic OR kinesitherapeutic OR therapeutic exercise OR exertion OR detraining)<br>#4 #1 AND #2 AND #3	
arXiv	Abstract=(Cancer OR Neoplas* OR Carcinoma OR Tumo* OR Adenocarcinoma OR Malignan*)<br>#2 Abstract=(Virtual reality)<br>#3 Abstract=(Virtual Reality Exposure Therapy OR Exergaming OR Exercise Therapy OR Exercise OR Sports OR Rehabilitation Exercise* OR Remedial Exercise* OR Exercise* OR Athletic* OR Training OR Active-Video Gaming* OR Exergam*)<br>#4 #1 AND #2 AND #3	1
IEEE Xplore	#1 (Mesh_Terms="Neoplasms") OR (Abstract="Cancer" OR "Neoplas*" OR "Carcinoma" OR "Tumo*" OR "Adenocarcinoma" OR "Malignan*")<br>#2 Abstract="Virtual Reality"<br>#3 Abstract="Virtual Reality Exposure Therapy" OR "Exergaming" OR "Exercise Therapy" OR "Exercise" OR "Sports" OR "Active-Video Gaming*" OR "Exergam*" OR "Rehabilitation Exercise*" OR "Remedial Exercise*" OR "Exercise*" OR "Athletic*" OR "Training"<br>#4 #1 AND #2 AND #3	36
MedRxiv	#1 Title=("Cancer" OR "Neoplas*" OR "Carcinoma" OR "Tumo*" OR "Adenocarcinoma" OR "Malignan*")<br>#2 Full Text or Title or Abstract=("Virtual Reality")<br>#3 Title or Abstract=("Virtual Reality Exposure Therapy" OR "Exercise" OR "Active-Video Gaming*" OR "Exergam*" OR	227

	"Athletic*" OR "Training")	
	#4 #1 AND #2 AND #3	

Table 2: Search Terms used of Chinese database

Database	Search Terms	Hits
CNKI	TKA='肿瘤' + '癌' AND SU='虚拟现实' AND TKA='运动疗法' + '运动康复' + '锻炼' + '训练'	15
Wanfang Data	题名或关键词=("肿瘤" OR "癌") and 主题=("虚拟现实") and 题名或关键词=("运动疗法" OR "运动康复" OR "锻炼" OR "训练")	11
VIP	M=(肿瘤 OR 癌) AND M=(虚拟现实) AND M=(运动疗法 OR 运动康复 OR 锻炼 OR 训练)	3
SinoMed	("肿瘤"[摘要:智能] OR "癌"[摘要:智能]) AND ("虚拟现实"[摘要:智能]) AND ("运动疗法"[摘要:智能] OR "运动康复"[摘要:智能] OR "锻炼"[摘要:智能] OR "训练"[摘要:智能])	22

Selection of studies

All search results were exported to Endnote X9, and screened and eliminated the duplication manually with it.

Study selection process started in two steps.

- First, we eliminated the irrelevant studies by reading titles and abstracts.
- Then, reading the full text of the remaining studies to determine the included studies by the same 2 researchers.

Data extraction and results synthesis

The extracted content included 4 parts. Tables 3 present example table shells.

- Metadata (author, year, country, study type, groups, analyzed sample size and

age)

- Features of VRER used (cancer type, existing or potential CRDs, VR model and device, intervention programs and duration)
- Measurement indictors and outcomes (effective results, compliance, patients` satisfaction and adverse events)

In this form, compliance, satisfaction rate or scores were either explicitly stated by the authors or calculated from flow charts.

Two researchers (Su and Lian) independently performed data extraction and cross-checking. If the opinions differed, the search terms were decided by another professor.

Table 3. Summary table shell example for clinical studies (study details)

Included studies (author, year, country)	Study type	Groups	Sample size, n (IG[a] /CG[b])	Age, years old, M±SD[c] (IG[a] /CG[b])	Cancer type	Type of existing or potential CRDs[d]	Types of VR[e]	VR[e] device	Program of VRER[f]	Duration of VRER[f]	Measurement indicators	Results (pre-/ control vs intervention group)	Compliance of VR[e] (%)	Satisfaction rate or scores of VR[e] system	Adverse events of VR[e] system

[a]IG: intervention group

[b]CG: control group

[c]M±SD: mean ± standard deviation

[d]CRDs: cancer-related dysfunctions

[e]VR: virtual reality

[f]VRER: virtual reality-based exercise rehabilitation

Results

The literature search was planned to be undertaken around September to October 2023.

Discussion

The scoping review would provide further information on the suitable type of VR device for VRER, VRER intervention methods, and the effect and patient attitudes. As healthcare evolves and technology advances, electronic interventions such as VR have become increasingly popular tools to support clinical management. The chronic development of cancer drives the digitalization of dysfunction management. Since 2013, VRER has begun to develop rapidly in CRDs, and the main studies are RCTs, aiming to study its effects and safety. While the number of VRER interventions in cancer patients is gradually increasing, it is important to consider the use of VRER and the effectiveness of the intervention to guide future development. By collating current research researchers can determine how VRER is being used in intervention and the current limitations. Furthermore, the scoping review identifies promising research and current research gaps that require further investigation.

Estimated Time Table

Task/ Time	W1	W2	W3	W4	W5	W6	W7	W8	W9	W10	W11	W12	W13	W14	W15	W15	W17	W18	W19	W20
Writing the protocol																				
Searching databases																				
Study selection																				
Data extraction																				
Data synthesis																				
Writing up																				

Author contributions

The review topic was conceived by Zhenzhen Su. The protocol was written by Zhenzhen Su, with final revision by Liyan Zhang, Miaomiao Guan and Xuemin Lian.

Funding

This project was supported by Scientific Research Foundation of Beijing Cancer Hospital, No KC2308. The sponsors had no role in the design and conduct of the study; preparation, review, or approval of the manuscript; and decision to submit the manuscript for publication.
