# Supplementary material for: Virtual Reality–Based Exercise Rehabilitation in Cancer-Related Dysfunctions: Scoping Review
Source: J Med Internet Res. 2024 Feb 26;26:e49312. doi: 10.2196/49312 (PMC10928524; doi:10.2196/49312)
Supplement: Multimedia Appendix 3 [file jmir_v26i1e49312_app3.pdf]

Multimedia Appendix 3. Search terms used to identify studies.

Database	Search Terms	Hits
PubMed	#1 "Neoplasms"[MeSH] OR "Cancer"[Title/Abstract] OR "Neoplas*"[Title/Abstract] OR "Carcinoma"[Title/Abstract] OR "Tumo*"[Title/Abstract] OR "Adenocarcinoma"[Title/Abstract] OR "Malignan*"[Title/Abstract]<br>#2 "Virtual Reality"[MeSH] OR ("Virtual"[Title/Abstract] AND "Reality"[Title/Abstract])<br>#3 "Virtual Reality Exposure Therapy"[MeSH] OR "Exergaming"[MeSH] OR "Exercise Therapy"[MeSH] OR "Exercise"[MeSH] OR "Sports"[MeSH] OR ("Reality Therap*"[Title/Abstract] AND "Virtual"[Title/Abstract]) OR "Active-Video Gaming*"[Title/Abstract] OR "Exergam*"[Title/Abstract] OR "Rehabilitation Exercise*"[Title/Abstract] OR "Remedial Exercise*"[Title/Abstract] OR "Exercise*"[Title/Abstract] OR "Athletic*"[Title/Abstract] OR "Training"[Title/Abstract]<br>#4 #1 AND #2 AND #3	249
Embase	#1 ‘malignant neoplasms’/exp OR ‘cancer’:ti,ab,kw OR ‘neoplas*’:ti,ab,kw OR ‘carcinoma’:ti,ab,kw OR ‘tumo*’:ti,ab,kw OR ‘adenocarcinoma’:ti,ab,kw OR ‘malignan*’:ti,ab,kw<br>#2 ‘virtual reality’/exp<br>#3 ‘exergaming’/exp OR ‘athletic rehabilitation’/exp OR ‘exercise’/exp OR ‘training’/exp OR ‘kinesiotherapy’/exp OR ‘active video gaming’:ti,ab,kw OR ‘exer-gaming’:ti,ab,kw OR ‘virtual reality-based exercise’:ti,ab,kw OR ‘sport rehabilitation’:ti,ab,kw OR ‘corrective exercise’:ti,ab,kw OR ‘exercise therapy’:ti,ab,kw OR ‘exercise movement techniques’:ti,ab,kw OR ‘exercise treatment’:ti,ab,kw OR ‘kinesiotherapeutic’:ti,ab,kw OR ‘kinesitherapeutic’:ti,ab,kw OR ‘therapeutic exercise’:ti,ab,kw OR ‘exertion’:ti,ab,kw OR	73

	'detraining':ti,ab,kw<br>#4 #1 AND #2 AND #3	
Scopus	#1 TITLE-ABS-KEY("Cancer" OR "Neoplas*" OR "Carcinoma" OR "Tumo*" OR "Adenocarcinoma" OR "Malignan*")<br>#2 TITLE-ABS-KEY("Virtual Reality" OR ("Virtual" AND "Reality"))<br>#3 TITLE-ABS-KEY("Virtual Reality Exposure Therapy" OR ("Reality Therap*" AND "Virtual") OR "Exergaming" OR "Exercise Therapy" OR "Exercise" OR "Sports" OR "Rehabilitation Exercise*" OR "Remedial Exercise*" OR "Exercise*" OR "Athletic*" OR "Training" OR "Active-Video Gaming*" OR "Exergam*")<br>#4 #1 AND #2 AND #3	663
Cochrane	#1 (Neoplasms)/exp OR (Cancer):ti,ab,kw OR (Neoplas*):ti,ab,kw OR (Carcinoma):ti,ab,kw OR (Tumo*):ti,ab,kw OR (Adenocarcinoma):ti,ab,kw OR (Malignan*):ti,ab,kw<br>#2 (Virtual Reality)/exp OR ((Virtual):ti,ab,kw AND (Reality):ti,ab,kw)<br>#3 (Virtual Reality Exposure Therapy)/exp OR (Exergaming)/exp OR (Exercise Therapy)/exp OR (Exercise)/exp OR (Sports)/exp OR ((Reality Therap*):ti,ab,kw AND (Virtual):ti,ab,kw) OR (Active-Video Gaming*):ti,ab,kw OR (Exergam*):ti,ab,kw OR (Rehabilitation Exercise*):ti,ab,kw OR (Remedial Exercise*):ti,ab,kw OR (Exercise*):ti,ab,kw OR (Athletic*):ti,ab,kw OR (Training):ti,ab,kw<br>#4 #1 AND #2 AND #3	198
Web of Science	#1 TS=(Cancer OR Neoplas* OR Carcinoma OR Tumo* OR Adenocarcinoma OR Malignan*)<br>#2 TS=(Virtual Reality OR (Virtual AND Reality))<br>#3 TS=(Virtual Reality Exposure Therapy OR (Reality Therap* AND Virtual) OR Exergaming OR Exercise Therapy OR Exercise OR Sports OR Rehabilitation Exercise* OR Remedial Exercise* OR Exercise* OR	1095

	Athletic* OR Training OR Active-Video Gaming* OR Exergam*)<br>#4 #1 AND #2 AND #3	
ProQuest	SU(malignant neoplasms) OR TI,AB(cancer OR neoplas* OR carcinoma OR tumo* OR adenocarcinoma OR malignan*)<br>#2 SU(virtual reality)<br>#3 SU(exergaming OR athletic rehabilitation OR exercise OR training OR kinesiotherapy) OR TI,AB(active video gaming OR exer-gaming OR virtual reality-based exercise OR sport rehabilitation OR corrective exercise OR exercise therapy OR exercise movement techniques OR exercise treatment OR kinesiotherapeutic OR kinesitherapeutic OR therapeutic exercise OR exertion OR detraining)<br>#4 #1 AND #2 AND #3	104
arXiv	Abstract=(Cancer OR Neoplas* OR Carcinoma OR Tumo* OR Adenocarcinoma OR Malignan*)<br>#2 Abstract=(Virtual reality)<br>#3 Abstract=(Virtual Reality Exposure Therapy OR Exergaming OR Exercise Therapy OR Exercise OR Sports OR Rehabilitation Exercise* OR Remedial Exercise* OR Exercise* OR Athletic* OR Training OR Active-Video Gaming* OR Exergam*)<br>#4 #1 AND #2 AND #3	1
IEEE Xplore	#1 (Mesh_Terms="Neoplasms") OR (Abstract="Cancer" OR "Neoplas*" OR "Carcinoma" OR "Tumo*" OR "Adenocarcinoma" OR "Malignan*")<br>#2 Abstract="Virtual Reality"<br>#3 Abstract="Virtual Reality Exposure Therapy" OR "Exergaming" OR "Exercise Therapy" OR "Exercise" OR "Sports" OR "Active-Video Gaming*" OR "Exergam*" OR "Rehabilitation Exercise*" OR "Remedial Exercise*" OR "Exercise*" OR "Athletic*" OR "Training"<br>#4 #1 AND #2 AND #3	36
MedRxiv	#1 Title=("Cancer" OR "Neoplas*" OR "Carcinoma" OR "Tumo*" OR	227

	"Adenocarcinoma" OR "Malignan*")<br>#2 Full Text or Title or Abstract=("Virtual Reality")<br>#3 Title or Abstract=("Virtual Reality Exposure Therapy" OR "Exercise" OR "Active-Video Gaming*" OR "Exergam*" OR "Athletic*" OR "Training")<br>#4 #1 AND #2 AND #3	
CNKI	TKA='肿瘤' + '癌' AND SU='虚拟现实' AND TKA='运动疗法' + '运动康复' + '锻炼' + '训练'	15
Wanfang Data	题名或关键词=("肿瘤" OR "癌") and 主题=("虚拟现实") and 题名或关键词=("运动疗法" OR "运动康复" OR "锻炼" OR "训练")	11
VIP	M=(肿瘤 OR 癌) AND M=(虚拟现实) AND M=(运动疗法 OR 运动康复 OR 锻炼 OR 训练)	3
SinoMed	("肿瘤"[摘要:智能] OR "癌"[摘要:智能]) AND ("虚拟现实"[摘要:智能]) AND ("运动疗法"[摘要:智能] OR "运动康复"[摘要:智能] OR "锻炼"[摘要:智能] OR "训练"[摘要:智能])	22
