# Supplementary material for: Virtual Reality–Based Exercise Rehabilitation in Cancer-Related Dysfunctions: Scoping Review
Source: J Med Internet Res. 2024 Feb 26;26:e49312. doi: 10.2196/49312 (PMC10928524; doi:10.2196/49312)
Supplement: Multimedia Appendix 4 [file jmir_v26i1e49312_app4.pdf]

Multimedia Appendix 4. Data extracted from all 25 studies.

Author, year, country	Study type	Groups	Sample size, n (IG[a] /CG[b])	Age (years), mean (SD[c]) or mean (range)	Cancer type	Type of existing or potential CRDs[d]	Types of VR[e]	VR device	Program of VRER[f]	Duration of VRER	Measurement indicators	Results (pre-/ control vs intervention group)	Compliance of VR (%)	Satisfaction rate or scores of VR system	Safety of VR system
Atef et al [35], 2020, Egyptian	Quasi-RCT[g]	VR group/ Proprioceptive neuromuscular facilitation group	15/15	54.1±8.3/ 53.1±7.2	Breast cancer	Postmastectomy lymphedema	Non-immersive VR	Nintendo Wii	After a short warm-up, the patients practiced a 30-min exercise program, including tennis, triceps extension, and rhythmic boxing using a VR device. One minute break was taken during the sessions between games.	Two sessions per week for 4 weeks.	Excess arm volume, upper limb function	Compared with pre-intervention, there were significant improvements in e excess arm volume and the affected upper limb dysfunction (9655.27 vs 6854.23; 55 vs 38, respectively, both P < 0.05). The percent improvement of excess arm volume was 26.47%, while the affected upper limb dysfunction improved by 33.66%.	83 (15/18)	NR[h]	NR
Basha et al [36], 2022, Saudi Arabia	RCT	Xbox Kinect group/ Resistance exercise group	30/30	48.8±7.0/ 52.1±7.5	Breast cancer	Postmastectomy lymphedema	Non-immersive VR	Xbox Kinect	"Macarena" dance and other Xbox Kinect games like darts, bowling, boxing, table tennis, fruit ninja, and beach volleyball.	Once per day, 5 times a week for 8 weeks.	Lymphedema symptom severity, physical function, and QoL[i]	Compared with pre-intervention, there were significant improvements in excessive limb volume by -2.63%, disability of arm, shoulder and hand by -14.7, ROMj by 25.83°, strength by 1.28kg, general health by 6.07, mental health by 4.53, vitality by 5.03, physical functioning by 14.7, social functioning by 2.77 and pain by -31.0. All P < 0.05. Compared with control group, there	100 (30/30)	NR	NR

												were significant improvements in pain intensity (53.76 vs 40.33), disability of arm (27.1 vs 22.9), shoulder ROM (including flexion, abduction and external rotation ROM, with the scores of 126.0 vs 139.0; 102.1 vs 123.3; 66.1 vs 75.0, respectively), general health (54.9 vs 58.4), and vitality (53.5 vs 55.7). All P < 0.05.			
Benzing, et al [37], 2020, Switzerland	RCT	Exergaming group/ Working memory training/CG	22/23/24	11.8±2.4/ 10.7±2.5/11.1±2.5	Various types (leukemia, lymphomas and nervous system cancers)	Central neuropathy due to radiotherapy or chemotherapy	Non-immersive VR	Xbox Kinect	Six different “workouts” incorporating physical (strength, coordination, and endurance) and cognitive demands. Such as “Waterfall Jump” (jumps onto wood without falling down) and “Derby Skate” (like dance, remember movements and imitates).	Three times a week for approximately 45 min, for 8 weeks.	Core executive functions, other cognitive domains, motor abilities, and parent rating on their children’s executive function	Linear mixed models revealed that no intervention effects of VRER could be detected (P > 0.05).	91 (20/22)	NR	NR
Chen et al [38], 2019, China	Before-after study in the same patient	NA[k]	80	NR	Breast cancer	CRCI[l]	Non-immersive VR	Self-built VR system	Allow patients to follow avatars to perform “Tai Chi”, “Cake Cutting”, “Rowing”, “Virtual shopping” and other trainings. For patients who can complete the training three times in a row, the system will give rewards; for patients who fail to complete, repeated training is required.	Once per day, 5 times a week for 8 weeks.	Cognitive function and QoL	Compared with pre-intervention, there were significant improvements in cognitive function (26.22 vs 17.41) and daily life ability (16.05 vs 20.79), all P < 0.05.	98 (78/80)	100 (80/80)	NR
da Silva Alves et al [40],	Before-after study in the same patient	NA	45	59.0±12.3	Various types (gastrointestinal tract,	CRF[m]	Non-immersive VR	Xbox Kinect	It is one part of a study. Including 3 games totally: “Wall Breaker” (to hit cubes projected), “Stomp	About 45 minutes per session, 3	CRF, strength of the medial	Compared with pre-intervention, there were significant	82 (45/55)	NR	NR

2017, Brazil					breast, Abdominal and pelvic and oropharyngeal cancer)				It" (hit lights by performing anterior and lateral movements of the lower limbs) and "Run the World" (to simulate walking using knee flexion and hip turn movements). "Stomp It" and "Wall Breaker" were required to practice for the first 10 sessions of the 20 sessions treatment, while all three were required to practice for the first 10 sessions.	sessions per week, for 8 weeks.	gastrocnemius, lateral gastrocnemius, and anterior tibialis muscles of both lower limbs	improvements in maximal voluntary isometric contraction of right dorsiflexor muscles (6.62 vs 10.9), right planter flexion (15.01 vs 22.59), and the left medial gastrocnemius (158.61 vs 185.23). All $P < 0.05$.			
da Silva Alves et al [39], 2018, Brazil	Before-after study in the same patient	NA	45	59.0±12.3	Various types (gastrointestinal tract, breast, Abdominal and pelvic and oropharyngeal cancer)	CRF	Non-immersive VR	Xbox Kinect	It is the other part of study with the different outcomes. Including 3 games totally: "Wall Breaker" (to hit cubes projected), "Stomp It" (hit lights by performing anterior and lateral movements of the lower limbs) and "Run the World" (to simulate walking using knee flexion and hip turn movements). "Stomp It" and "Wall Breaker" were required to practice for the first 10 sessions of the 20 sessions treatment, while all three were required to practice for the first 10 sessions.	About 45 minutes per session, 3 sessions per week, for 8 weeks.	QoL, CRF	Compared with pre-intervention, there were significant improvements in the general functional assessment (88.13 vs 95.37), functional outcomes index (76.00 vs 92.68), and fatigue assessment (121.20 vs 138.99). All $P < 0.05$.	82 (45/55)	NR	NR
Feyzioĝlu et al [41], 2020, Turkey	RCT	Kinect-based rehabilitation group/ Standardized physical therapy group	20/20	50.8±8.5/ 51.0±7.1	Breast cancer	Postmastectomy upper limb dyskinesia	Non-immersive VR	Xbox Kinect	After 5 min warm-up, darts, bowling and boxing were played for the first 3 weeks of the 6 weeks treatment, while beach volleyball, table tennis and "Fruit Ninja" were played for last 3 weeks.	The treatment for 45 minutes per session and two times a week for 6 weeks.	Pain intensity, ROM, shoulder muscle strength, hand grip strength, upper extremity functionality, ROM, and fear of movement	Compared with pre-intervention, there were significant improvements in pain (6.53 vs 1.53), ROM by 55.39°, muscle strength (5.97 vs 6.25), grip strength (21.47 vs 23.71), functionality (44.67 vs 16.49), and fear scores (42.37 vs 29.47). All $P < 0.05$. Compared with control group, fear of movement was	95 (19/20)	NR	NR

												significantly improved by VRER, while the traditional exercise rehabilitation displayed more improvement in functionality (5.94 vs 12.9, -36.54 vs -28.18, respectively). Both P < 0.05.			
Hamari et al [42], 2019, Finland	RCT	IG/CG	17/19	7.8 (range, 3-16)/ 7.9 (range, 3-15)	Various types (Wilms' cancer, acute lymphocytic leukemia and lymphoma)	Physical function damage, CRF	Non-immersive VR	Nintendo Wii	Nintendo Wii games	Thirty minutes a day, 7 days a week for 8 weeks	Physical activity, the metabolic equivalent, motor performance, fatigue, experiences and fidelity of the intervention, and acceptability of the intervention	No significant difference (P > 0.05).	71 (12/17)	NR	NR
Hoffman et al [43], 2013, America	Before-after study in the same patient	NA	7	64.6 (range, 53–73)	Lung cancer	CRF	Non-immersive VR	Nintendo Wii	It is the first stage of study. Intervention consisted of two parts, one was walking with the Wii, the other is balance exercise including downhill skiing, soccer, golf, and video game activities.	Thirty minutes a day, 5 days a week, for 6 weeks. It was anticipated to increase by 5 minutes per day with the goal of walking 30 minutes per day during week 6.	Feasibility, acceptability, safety of VRER, CRF, self-management of fatigue, walking, and balance, CRF self-management behaviors, and functional performance	Compared with pre-intervention, there were improvements in CRF severity (4.8 vs 2.5); perceived self-efficacy for fatigue self-management (7 vs 8.8), walking and balance (47.4% vs 93.3%, 72.8% vs 83.7%, respectively); and functional performance (steps taken per day, 4650 vs 6393). No statistical analysis.	100 (7/7)	Scores 5.8±0.18 of 6	None
Hoffman	Before-after	NA	7	64.6 (range, 53–	Lung cancer	CRF	Non-	Nintendo	It is the second stage of the study,	Thirty	Feasibility,	Compared with pre-	88 (study	Scores	None

et al [44], 2014, America	study in the same patient			73)			immersive VR	Wii	and was a continuation of phase I. VRER: the home-based intervention promoted light-intensity, less than 3.0 metabolic equivalents, walking, and balance exercises utilizing VR system.	minutes a day, 5 days a week, for another 10 weeks.	acceptability, safety of VRER, CRF, self-management of fatigue, walking, and balance, CRF self-management behaviors, and functional performance	intervention, there were improvements in CRF severity (4.8 vs 1.3); perceived self-efficacy for fatigue self-management (7 vs 9), walking and balance (47.4% vs 99.4%, 72.8% vs 88.9%, respectively); and functional performance (steps taken per day, 4650 vs 7683). No statistical analysis.	reported)	5.8±0.17 of 6	
House et al [45], 2016, America	Before-after study in the same patient	NA	6	57.8±20.4	Breast cancer	CRCI, postmastectomy upper limb dyskinesia	Non-immersive VR	Self-built VR system	Nine custom games: "Breakout 3D" (bounce a virtual ball by paddle avatars), "Card Island" and "Remember that Card" (match card), "Musical Drums" (strike notes using drum stick avatars), "Xylophone" game (repeat notes using mallet avatars), "Pick & Place" (grasp and move balls), "Arm Slalom" (rotate shoulder to guide a skier avatar through a downhill slalom), "Avalanche" (control a pick axe and a shovel avatar to break and clear ice walls), "Treasure Hunt" game (control shovel avatars to find buried treasures)	Twenty to 50 minutes per sessions, 2 sessions per week for 8 weeks.	Feasibility of the VR system, pain, cognition, muscle strength, ROM, activity of daily life	Compared with pre-intervention, there were significant improvements in ROM by 8°, the strength by 8.2N, The daily life ability by 13.8, the depression by -5.7, the visuospatial memory by 8. All P < 0.05.	67 (6/9)	Scores 4.83 of 5	NR
Jin et al [47], 2018, China	Quasi-RCT	IG/CG	38/38	Range from 33 to 36/ range from 35 to 69	Breast cancer	Postmastectomy upper limb dyskinesia	Non-immersive VR	Self-built VR system	Process was divided into four phases. Phase I (1~7 days after surgery): strength finger, hold and squeeze balls, knead and fingertip. Phase II (8~14 days after surgery): move like a pendulum shrug, climb wall, move upper limb and spiral arm. Phase III (15 days~1 month after	Fifteen to 30 minutes per session, 2 sessions per day for 3 months	ROM, compliance, edema	Compared with control group, there were significant improvements in ROM of shoulder (57.8°vs 81.6°), compliance (84% vs 100%), edema rete of affected limbs (42%	100 (38/38)	NR	NR

									surgery): flying, swing rope, throw ball, exercise with dumbbell and elastic band. Phase IV (1~3 months after surgery): warm-up, shake hand, extend arm, turn waist and circle.			vs 11%). All P < 0.05.			
Jin et al [46], 2018, China	RCT	VR group/ Conventional rehabilitation group	60/60	NR	Breast cancer	Postmastectomy upper limb dyskinesia	Non-immersive VR	Self-built VR system	Exercises with VR, including breath and raise leg, throw ball, comb hair, pendulum, shrug shoulder, rotate arm, raise hand, expanse chest, jump, rollers.	Thirty minutes per sessions, 1 session per day, 6 days per week, for 3 months.	QoL	Compared with control group, there was significant improvement in QoL (75.1 vs 85.4, P < 0.05).	NR	NR	NR
Lin et al [48], 2021, China	RCT	IG/CG	68/68	33.0±2.8/ 33.1±2.8	Breast cancer	CRCI	Non-immersive VR	Self-built VR system	Virtual cognitive rehabilitation training consists of 4 phages. Phase 1 (1 week after surgery), it included finger stretching, ball squeezing, ball holding, and fingertip rubbing. Phase 2 (1 to 2 weeks after surgery), as moving the upper arms, relaxing the shoulders and neck, rotating the body, and lifting shoulders, supplemented by pendulum and shoulder shrugging, wall climbing, arm swing and other exercises. Phase 3 (3 to 4 weeks after surgery), including contraction, lateral push and pull, hand swing, chest expansion, lateral lift, circumference, abdominal and back muscle training, lifts, body turns, and others. Phase 4 (5 to 8 weeks after surgery), such as head shaking, arm extension, side waist turning, circle exercises.	For 8 weeks	The incidence of complications, compliance, shoulder joint activity, activities of daily living and QoL, cognitive function	Compared with control group, there were significant improvements in postoperative complications rates (67% vs 11%), compliance (71% vs 95%), ROM of shoulder (57.9° vs 81.6°), daily life ability (20.84 vs 16.07), QoL (15.39 vs 18.69) and cognition (24.60 vs 29.46). All P < 0.05.	93(63/68)	NR	NR
Park et al [49], 2023, Korea	RCT	UINCARE Home+ group/ Brochure-	50/50	42.6±9.1/ 47.3±8.6	Breast cancer	Postmastectomy syndrome	Augmented reality	UINCARE Home+	Augmented reality exercise programs consisted of 2 parts with 4 level. Part 1 included passive and active forward	Thirty minutes with 12~14 exercises	ROM and pain of affected shoulder,	Compared with pre-intervention, there were significant improvements in	94 (47/50)	Scores 3.28 of 4	None

		based home rehabilitation group							flexion, external rotation and abduction, and trunk rotation with both shoulder forward flexion. Part 2 added exercise with dumbbell and pectoralis stretching in addition to part 1.	per session, 5 sessions per day, 7 days per week, for 8 weeks.	upper limb function, QoL	ROM (109.56 vs 169.12), limb dysfunction (22.05 vs 17.07) and QoL (0.69 vs 0.85). All P < 0.05. Compared with control group, no significant difference (P > 0.05).			
Sabel et al [50], 2016, Sweden	RCT	IG/CG	7/6	11.9±3.6/ 13.2±1.9	Brain cancer	Imbalance because of central neuropathy	Non-immersive VR	Nintendo Wii	Games were used including “Sports”, “Sports Resort”, “Fit”, “Fit plus”, “Dance”, “Michael Jackson Dance”.	Thirty minutes per day, 5 days per weeks for 10 weeks	Physical level and function, compliance	Compared with pre-intervention, the Body Coordination score improved by 15% (P < 0.05). Compared with control group, no significant difference (P > 0.05).	100 (13/13)	87% (6/7)	NR
Schwenk et al [51], 2016, America	RCT	IG/CG	11/11	68.7±8.7/ 71.8±8.9	Various types (multiple myeloma, chronic lymphoid leukemia, lung, breast, colorectal, melanoma, bladder, prostate, pancreas, and ovarian cancer)	Chemotherapy-induced peripheral neuropathy	Non-immersive VR	Self-built VR system	Three tasks. Balance exercises: forward/backward/sideward/diagonal leaning tasks. Ankle point-to-point reaching task: forward/backward/sideward/diagonal leaning and partial weight transfer. Virtual obstacle crossing task: crosse obstacles	Forty-five per sessions, two sessions per week, for 4 weeks.	Balance, gait performance, fear of falling	Compared with control group, here were significant improvements in medial-lateral center of mass sway (21.2% vs 55.5%), hip sway (42.3% vs 67.5) and ankle sway (21.4% vs 68.2%). All P < 0.05.	82 (9/11)	NR	NR
Tanriverdi et al [52], 2022, Turkey	Before-after study in the same patient	NA	11	12.9±5.8	Acute lymphoblastic leukemia	Sleep disorder	Non-immersive VR	Nintendo Wii	The virtual reality-based games were free jogging, hula hoop, step, and step plus.	Forty-five minutes per session, 2 sessions per day, 2 days per week, for 12 weeks.	Sleep condition	Compared with pre-intervention, there were significant improvements in Respiratory Disturbance Index (3.10 vs 1.97), number of apnea (15.55 vs 10.14), and children`s sleep habits	100 (11/11)	NR	NR

												(57.45 vs 48.27). All P < 0.05.			
Tsuda et al [53], 2016, Japan	Before-after study in the same patient	NA	16	66.0±5.4	Lymphoma, acute leukemia	Physical function damage	Non-immersive VR	Nintendo Wii	Two activities: “Hula Hoop” and “Basic Step” were played of light to moderate intensity.	Twenty minutes per session, once per day, 5 times a week, from the start of chemotherapy until hospital discharge	Adherence rate, safety, physical and psychological performance	Compared with pre-intervention, no significant difference (P > 0.05).	56 (9/16)	NR	None
Villumsen et al [54], 2019, Denmark	RCT	Unsupervised home-based exergaming group/ Usual care group	23/23	67.6±4.6/ 69.8±4.4	Prostate cancer	Physical function damage	Non-immersive VR	Xbox Kinect	Aerobic and strength exercise for 1 hour using “Your Shape Fitness Evolved 2012”, “Sport” and “Adventure” games.	About 1 hour per session, three sessions a week for 12 weeks	Six-min walking test, leg extensor power, body composition, self-reported physical functioning and global health status, QoL, CRF	Compared with control group, here was significant improvement in the six-min walking test by 4.2% (P < 0.05).	91 (21/23)	NR	None
Yang et al [55], 2014, Korea	RCT	VR group/ CG	19/19	47.9±14.5/ 52.9±14.0	Brain cancer	CRCI	Non-immersive VR	IREX system	Five programs were used: “Conveyor” (move boxes from one side to the other), “Coconut” (catch and coconuts into a basket), “Bird and Balls” (catch the birds), “Soccer” (stop the balls from entering the net), “Juggler” (hit the balls)	Thirty minutes a day, 3 times a week, for 4 weeks	Cognitive function, functional status	Compared with pre-intervention, there were significant improvements in continuous performance test (0.65 vs 0.55), digit span test (3.9 vs 5.1), visual span test (3.5 vs 4.6), learning test-recognition (30.5 vs 40.7), Trail Making Test-type A (137.9 vs 70.2), Korean version of Modified Barthel Index (43.4 vs 73.7) and Korean version of the Mini-Mental	100 (19/19)	NR	NR

												Status Examination (19.8 vs 25.0). All P < 0.05. Compared with control group, there were significant improvements in continuous performance test (-0.2 vs -0.0), backward digit span test (1.4 vs 0.3), backward visual span test (1.4 vs 0.7) and Trail Making Test-type A (-67.7 vs -24.5). All P < 0.5.			
Yoon et al [56], 2015, Korea	Non-RCT	IG/CG	20/20	48.6±11.3/ 50.0±17.5	Brain cancer	Postmastectomy upper limb dyskinesia	Non-immersive VR	IREX system	Six VR programs were selected: “Birds and Balls”, “Conveyor”, “Drums” (use hands to play a drum along with the music), “Juggler”, “Coconuts”, and “Soccer”.	Thirty minutes per day, 3 days per week for 3 weeks.	Upper-extremity function and activities of daily living	Compared with pre-intervention, here were significant improvements in manual dexterity (30.5 vs 38.0), Manual Function test (70.3 vs 82.8) and Fugl-Meyer scale (52.0 vs 58.0) of upper-extremity motor ability, activity of life (52.5 vs 70.5). All P < 0.05. Compared with control group, there were significant improvements in manual dexterity (8.0 vs 11.0), Manual Function test of (shoulder/elbow/forearm section, 5.0 vs 7.0), Fugl-Meyer scale (shoulder/elbow/fore	83 (20/24)	100% (20/20)	None

												arm section, 2.0 vs 3.5). All $P < 0.05$.			
Zeng et al [57], 2020, China	Observational feasibility study	NA	21	NR	NR	CRCI	Immersive VR	Self-built VR system	Whack-a-mole, golf, fruit cutting, shooting	NA	The safety of VR system	All subjects thought the all-in-one VR machine was safe.	NA	100% (21/21)	None
Zhou et al [58], 2021, China	Observational feasibility study	NA	15	54.7±7.8	Breast cancer	Postmastectomy upper limb dyskinesia	Immersive VR	Self-built VR system	Two models. Model 1 included make fist, rotate wrist and bend elbow for short term after surgery, while model 2 included make fists, rotate wrists, bend elbows, lift up, wrap shoulders, touch ears, climb walls, put hands behind your back, hold head and abduct for the long.	NA	Availability, adverse events, immersion	The VR system had high acceptance and availability, and patients had a high immersion during using, while had little impact of sickness.	NA	NR	Light sicknes s
Zhu et al [59], 2019, China	RCT	IG/CG	40/40	58.3±15.4/ 58.6±15.1	Breast cancer	Postmastectomy upper limb dyskinesia	Non-immersive VR	Self-built VR system	Four phases. Phase 1 (1 week after surgery): joint functional training like stretch fingers, squeeze and hold balls and knead fingertips. Phase 2 (2 weeks after surgery): exercises like shoulder joint abduction, adduction, extension and forward flexion exercises. Phase 3 (3~4 weeks after): resistance training with dumbbell. Phase 4 (1~3 months after): normal exercise with low intensity.	Thirty minutes per session, 2 sessions per day, for 3 months	ROM, adherence, complication	Compared with control group, there were significant improvements in compliance (72.5 vs 90), ROM of shoulder (57.84° vs 81.65°), and postoperative complication rate (12.5% vs 32.5%). All $P < 0.05$.	90 (36/40)	NR	NR

[a]IG: intervention group

[b]CG: control group

[c]SD: standard deviation

[d]CRDs: cancer-related dysfunctions

[e]VR: virtual reality

[f]VRER: virtual reality-based exercise rehabilitation

[g]RCT: randomized clinical trial

[h]NR: not reported

[i]QoL: quality of life

[j]ROM: range of motion

[k]NA: not available

[l]CRCI: cancer-related cognitive impairment

[m]CRF: cancer-related fatigue
